# [^18^F]Tosyl fluoride as a versatile [^18^F]fluoride source for the preparation of ^18^F-labeled radiopharmaceuticals

**DOI:** 10.1038/s41598-023-30200-2

**Published:** 2023-02-23

**Authors:** Dong Zhou, Wenhua Chu, Jinbin Xu, Sally Schwarz, John A. Katzenellenbogen

**Affiliations:** 1 Department of Radiology, School of Medicine, Washington University in Saint Louis, 510 S. Kingshighway Blvd, Saint Louis, MO 63110 USA; 2 Department of Chemistry and Cancer Center at Illinois, University of Illinois at Urbana-Champaign, Champaign, IL 61801 USA

**Keywords:** Nuclear chemistry, Positron-emission tomography, Process chemistry

## Abstract

Positron emission tomography (PET) is an in vivo imaging technology that utilizes positron-emitting radioisotope-labeled compounds as PET radiotracers that are commonly used in clinic and in various research areas, including oncology, cardiology, and neurology. Fluorine-18 is the most widely used PET-radionuclide and commonly produced by proton bombardment of ^18^O-enriched water in a cyclotron. The [^18^F]fluoride thus obtained generally requires processing by azeotropic drying in order to completely remove H_2_O before it can be used for nucleophilic radiofluorination. In general, the drying step is important in facilitating the radiofluorination reactions and the preparation of ^18^F-labeled PET radiotracers. In this communication, we have demonstrated the feasibility of using [^18^F]tosyl fluoride ([^18^F]TsF) as a versatile [^18^F]fluoride source for radiofluorination to bypass the azeotropic drying step, and we have developed a continuous flow solid-phase radiosynthesis strategy to generate [^18^F]TsF in a form that is excellent for radiofluorination. [^18^F]TsF shows high reactivity in radiofluorination and provides the features suitable for preparing PET radiotracers on a small scale and exploring novel radiolabeling technologies. Thus, using [^18^F]TsF as a [^18^F]fluoride source is a promising strategy that facilitates radiofluorination and provides a convenient and efficient solution for the preparation of ^18^F-labeled radiopharmaceuticals that is well matched to the emerging trends in PET imaging technologies.

## Introduction

Positron emission tomography (PET) is commonly used in pre-clinical and clinical studies for the in vivo imaging of—often trace levels of—biological targets and processes in humans or animals, utilizing positron emitter-labeled molecules (e.g. O_2_, water, sugars, amino acids, peptides, and various pharmaceuticals) as PET radiotracers^1^. Fluorine-18 is the most widely used radioisotope for PET for various reasons, including its favorable decay features and facile production with medical cyclotrons^2^. Fluorine-18 labeled compounds are commonly produced by nucleophilic substitution of suitable precursors using [^18^F]fluoride ion as a nucleophile^2,3^. [^18^F]Fluoride is usually produced in the quantity of nmol in a cyclotron by proton bombardment of an ^18^O-enriched water target via the ^18^O(p,n)^18^F nuclear reaction. The expensive [^18^O]water generally needs to be recovered for recycling, and the [^18^F]fluoride has to be dried (i.e., freed from fluoride ion hydration) so that it will dissolve in a suitable organic solvent in a form having sufficient nucleophilicity for subsequent substitution reactions^3^.

The common practice for drying [^18^F]fluoride includes recovering the [^18^O]water and trapping [^18^F]fluoride on an ion exchange resin cartridge, eluting [^18^F]fluoride from the cartridge with an aqueous solution of a selected base, and then azeotropic evaporation after continual addition of acetonitrile at around 100 °C under a gentle flow of inert gas (in some cases, vacuum is also applied) before dissolution in an organic solvent for further substitution reactions^4^. Remarkably, despite the many drawbacks noted below, this commonly used protocol has remained essentially unchanged for the past 40 years! The problems with the azeotropic drying step are the following: (a) A large amount of base (e.g., 1 mg potassium carbonate) is generally required to elute [^18^F]fluoride efficiently from the cartridge (e.g. Waters QMA 46 mg and Chromafix 30-PS-HCO_3_ 45 mg); this amount of base requires a correspondingly large amount of labeling precursor and can result in low yields for base-sensitive precursors/products, limiting the flexibility for altering reaction conditions. (b) The azeotropic drying also requires a complicated setup for an automated synthesis module, and thus is poorly suited for miniaturizing a radiolabeling reaction and adapting it for use in a synthesis module. (c) Impurities can be introduced from the anion exchange resin and in the drying step, and they may be detrimental to radiolabeling reactions. (d) Furthermore, the drying step is time-consuming, resulting in radioactivity loss due to decay, and (e) the dried [^18^F]fluoride may not be storable and thus not ideal for dose-on-demand radiosynthesis. Perhaps most important, (f) if the azeotropic drying is not done properly, it often results in low reproducibility or even failure of radiolabeling reactions, and this poor reliability and reproducibility in the production of PET radiotracers will limit their clinical and research uses. These problems often contribute to making the efficient and convenient preparation of ^18^F-labeled radiotracers challenging.

Because the azeotropic drying of [^18^F]fluoride is usually the essential step for radiofluorination and the hurdle for microfluidic chemistry, many efforts have been undertaken to develop methods capable of producing [^18^F]fluoride for radiolabeling without this azeotropic drying step^5–12^. Reported approaches include using anion exchange resins to trap/elute [^18^F]fluoride^13^, trapping and drying in an electrochemical cell^14^, and chemical conversions using ^18^F-labeled intermediates^13^. The trap/elution method is simple and compatible with automated modules as the current modules are designed for this, but it is limited to some applications; interested readers are referred to a paper with an extensive review of the recent advances of this methodology^15^. The electrochemical cell is rarely used in conventional radiolabeling. The chemical conversion approach, also termed as purification of [^18^F]fluoride^3^, takes advantage of the facile formation of ^18^F-labeled gaseous species (e.g. Me_3_SiF^16^, AcF^17^, TfF^18^, ethenesulfonyl fluoride^19^), which can be easily separated from the reaction mixture and then trapped and converted back to [^18^F]fluoride by a base for radiolabeling reactions. [^18^F]Fluoride generated by this approach showed superior reactivity, but these gaseous radioactive species are not practical to use and present safety concerns.

Among the ^18^F-labeled chemical species reported, ^18^F-labeled sulfonyl fluorides as a [^18^F]fluoride source have shown promise for the preparation of ^18^F-labeled radiotracers. This strategy includes the facile and exclusive formation of the sulfonyl fluoride and its quantitative conversion to reactive fluoride ion under basic conditions^20^, thereby allowing the smooth transformation of [^18^F]fluoride from the ^18^O water to a suitable anhydrous organic solvent for radiolabeling, without an explicit azeotropic drying step. A fast and reliable method for generating [^18^F]triflyl fluoride ([^18^F]TfF) as a gaseous [^18^F]fluoride source has been reported^18^, and we have developed a simple method to generate [^18^F]TfF by using an anion exchange resin cartridge to trap and dry [^18^F]fluoride, and then generate [^18^F]TfF directly on the same cartridge^21^. However, [^18^F]TfF does not provide a solution to overcome the aforementioned practicality and safety drawbacks. In this report, we describe the generation of [^18^F]tosyl fluoride ([^18^F]TsF) as a versatile and promising [^18^F]fluoride source and its application in the preparation of ^18^F-labeled PET radiotracers, demonstrating its great potential for facilitating the preparation of ^18^F-labeled radiotracers to meet the future needs of radiopharmaceuticals (RPs) for PET imaging in clinic and research.

## Results

**Figure 1:**
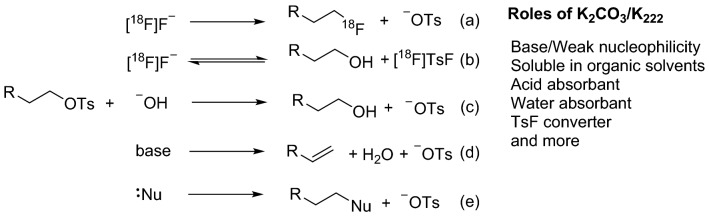
Conventional aliphatic nucleophilic substitution radiofluorination and its side-reactions. Besides the reactions with [^18^F]fluoride to form the desired product (**a**) and [^18^F]TsF (**b**), the tosylate precursor may also react with bases (e.g. hydroxide from K_2_CO_3_ and residual water) to form the alcohol (**c**) and the eliminated product (**d**) and with other nucleophiles (e.g. chloride from QMA cartridge and nucleophilic atom(s) on the precursor itself (**e**). Collectively, the complex of potassium carbonate/Kryptofix 222 plays multiple roles in maintaining the reaction conditions that lead to the formation of the desired radioactive product.

Sulfonates (e.g., tosylates) are the most widely used leaving groups in aliphatic nucleophilic substitution radiofluorination. Potassium carbonate as the base and Kryptofix® 222 as the phase transfer agent (K_2_CO_3_/K_222_) is the most used combination in these reactions due to its superior performance in radiolabeling. The potential roles of K_2_CO_3_/K_222_ in radiofluorination are discussed in Fig. 1. In a typical aliphatic nucleophilic substitution radiolabeling using a tosylate precursor, there are two substitution reactions occurring with [^18^F]fluoride: one is to form the desired fluoroalkyl product, which is less likely to be converted back to [^18^F]fluoride (reaction a); another one is the formation of [^18^F]TsF, which will be instantly converted back to fluoride under basic conditions for further reaction (reaction b). Actually, [^18^F]TsF has been observed as the major radioactive product under mild reaction conditions^22^. Collectively, K_2_CO_3_/K_222_ plays many important roles in nucleophilic radiofluorination reactions, maintaining the reaction conditions that lead to the formation of the desired radioactive product (Fig. 1). It is rational to use [^18^F]TsF with K_2_CO_3_/K_222_ as a [^18^F]TsF converter for radiofluorination.

### Optimizing the generation of [^18^F]sulfonyl fluorides

The synthesis of [^18^F]TsF from tosyl chloride has been reported as a model radiolabeling reaction under conventional radiolabeling conditions^23,24^. However, adapting [^18^F]TsF as a practical source of [^18^F]fluoride ion in radiofluorination reactions requires that it be generated rapidly, in high radiochemical yields, with high chemical and radiochemical purities, at high molar activity (or minimum amount of [^19^F]fluoride introduced during the process), and in small volumes of anhydrous solvents suitable for radiolabeling. None of these features were provided by the conventional radiosynthesis of [^18^F]TsF.

We demonstrated the facile conversion of [^18^F]fluoride to [^18^F]sulfonyl fluorides at room temperature using a variety of sulfonyl chlorides; notably, these reactions even took place in 1:1 acetonitrile/PBS buffer at pH 7.4 (Basic conditions were required for this conversion, supplementary Table S1). To generate [^18^F]TsF, tosyl chloride (TsCl) was used as a practical and convenient precursor because it is readily available in high purity (free from detectable levels of TsF), stable in neutral solvents, and UV visible for HPLC detection. We first attempted to use TsCl in acetonitrile to elute radioactivity from a 30-PS-HCO_3_ cartridge onto which [^18^F]fluoride has been trapped and dried by rinsing with acetonitrile. This resulted in the elution of over 90% of radioactivity as [^18^F]TsF; however, a significant amount of TsCl was present in the eluted solution (See Supplementary Figure. S2). The speed and efficiency of this preliminary try prompted us to explore modifications of this general approach to improve the purity but maintain a high yield in the production of [^18^F]TsF.

### Selecting the best solvent and resin

Various solvents were used for eluting the activity from the resin (Supplementary Table S2). Very high radiochemical conversions (RCCs) were observed in 2-amyl alcohol and ethanol, and this should be due to the facile release of the trapped [^18^F]fluoride ion from the resin by these alcohols for the reaction with TsCl. Among other commonly used solvents for radiofluorination, acetonitrile afforded over 90% RCC at room temperature or at 80 °C. In many respects, acetonitrile is an ideal solvent for the radiofluorination step in the preparation of ^18^F-labeled radiotracers; so, further exploration was done mainly using acetonitrile as the eluting solvent.

Different anion exchange resins were tested for RCCs (Supplementary Table S3). Polymer-based anion exchange resins gave high RCCs, whereas silica-based ones (Waters QMA) resulted in poor RCC, likely due to the direct interaction of fluoride ion with the silica matrix. The commercially available cartridge 30-PS-HCO_3_ gave the best results; so it was used for the further development of this method, along with its equivalent MP-1 M resin version, the use of which has been reported elsewhere for trapping [^18^F]fluoride^25^. Other sulfonyl derivatives were also tested for the efficiency with which they could be recovered as the corresponding [^18^F]sulfonyl fluorides (Supplementary Table S4), and the TsF/TsCl pair appeared to be the best choice.

### Removal of TsCl

The preceding studies showed that the elution of [^18^F]fluoride from an anion exchange resin using sulfonyl chlorides is a robust, convenient, and efficient method for obtaining [^18^F]sulfonyl fluorides. Various levels of sulfonyl chlorides, however, were co-eluted with the [^18^F]sulfonyl fluorides, ranging from 0.1 to over 50% of the starting sulfonyl chlorides. Because large amounts of sulfonyl chlorides will interfere with radiolabeling reactions, often resulting in reaction failures, these sulfonyl chlorides needed to be removed.

Since sulfonyl fluorides are much more stable than sulfonyl chlorides under basic conditions^26^; so, while they are in contact with basic anion exchange resins (HCO_3_^−^ ion and residual water), sulfonyl chlorides readily decompose to sulfonate and chloride ions, both of which have higher affinity and selectivity for the resin than [^18^F]fluoride ion. Thus, when the sulfonyl chloride is added to the column, fluoride ion trapped by the resin is released to react with the sulfonyl chloride, forming the more stable and neutral sulfonyl fluoride. The remaining, excess sulfonyl chlorides can then be removed while on the anion exchange resins by the aforementioned decomposition and trapping mechanism.

**Figure 2:**
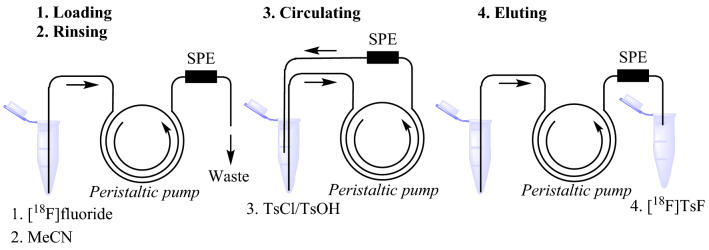
Manual generation of [^18^F]TsF using a peristaltic pump as a proof of concept of continuous flow solid-phase radiosynthesis of [^18^F]TsF. The steps include (**1**) loading [^18^F]fluoride onto the SPE cartridge and (**2**) drying the SPE cartridge using acetonitrile, (**3**) circulating a solution of TsCl/TsOH·H_2_O, and (**4**) eluting [^18^F]TsF from the SPE cartridge. For greater details, see “Methods” section.

When the distribution of trapped [^18^F]fluoride in the resins [30-PS-HCO_3_ (46 mg) and MP-1 M-HCO_3_ (30 mg)] was studied, we found that 80–90% radioactivity was trapped on the top layer of the cartridges. The eluted solution containing [^18^F]TsF and TsCl was reloaded onto the same cartridge to allow any leftover [^18^F]fluoride ion to react with leftover TsCl and, at the same time, to progressively remove any excess TsCl. At the same time, TsOH and HCl from the decomposition of TsCl will begin to neutralize the basic resin to minimize the decomposition of the [^18^F]TsF during this reloading. This process was aided by the addition of a small amount of TsOH H_2_O (0.25 mg) to the eluting solution of TsCl (1 mg) to improve the RCCs and reproducibility. The reloading procedure was achieved by using a peristaltic pump to circulate the eluting solution through the cartridge (Fig. 2). The optimized conditions are a flow rate at 3 mL/min for 3 min (Supplementary Table S5), as this resulted in the highest level of TsF formation and the complete elimination of TsCl.

**Figure 3:**
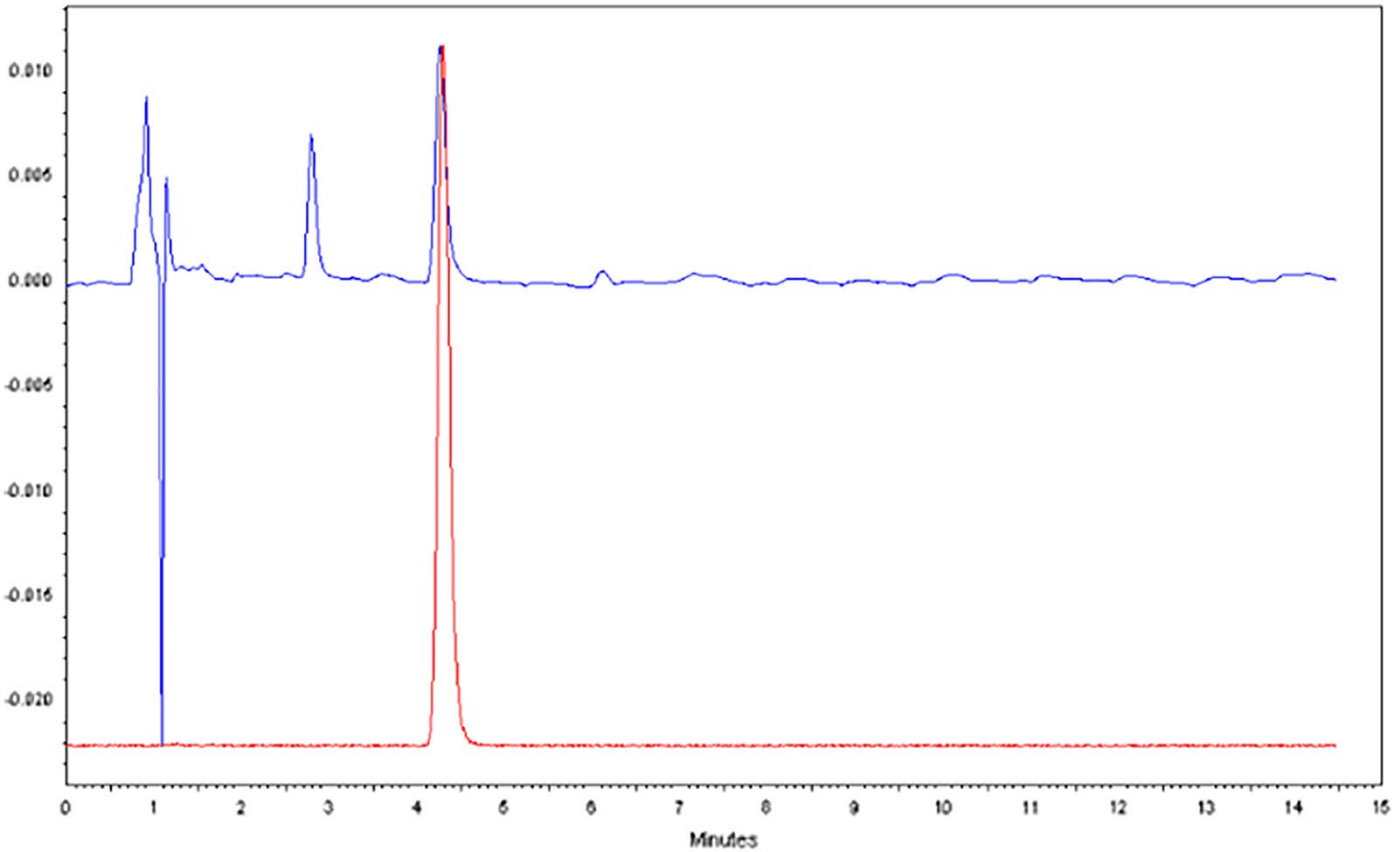
Analytical radio-HPLC of [^18^F]TsF, analyzed 12 h later after generation of [^18^F]TsF (Red: UV; Blue: radioactivity). The peak at 3 min is from TsCl and TsCl at 5.5 min was not observed.

Under these optimized conditions, [^18^F]TsF can be generated within 10 min in up to 97% RCCs, with 100% radiochemical purity (Fig. 3). The dead volume of the peristaltic pump is about 0.5 mL, so over 90% of the radioactivity can be collected in 1 mL of acetonitrile after the pump is rinsed with acetonitrile (0.5 mL). [^18^F]TsF is stable in acetonitrile, and the solution can be stored before use. [^18^F]TsF, with a melting point of 41–42 °C (TsF), is not volatile (Nevertheless, the processing of [^18^F]TsF should be carried out inside a fume hood), and its solution in acetonitrile can be transferred or dispatched easily using a pipette. Since TsF has high UV absorbance, the molar activity (A_m_) of [^18^F]TsF can be measured easily by analytical radio-HPLC analysis^27^. We have determined that only a tiny amount of [^19^F]fluoride was introduced from the resin (0.008 µg from 30 mg MP-1 M-HCO_3_ and 0.011 µg from 46 mg 30-PS-HCO_3_) and none from TsCl by HPLC. This is critical for the preparation of ^18^F-labeled radiotracers with high A_m_ when small amounts of ^18^F radioactivity are used.

### Considerations in the use of [^18^F]TsF to generate [^18^F]fluoride ion for radiofluorination

The conversion of [^18^F]TsF to [^18^F]fluoride can be achieved either in situ by heating a mixture of a precursor, [^18^F]TsF and K_2_CO_3_/K_222_ or by pre-heating the solution of [^18^F]TsF with K_2_CO_3_/K_222_ before the addition of a radiolabeling precursor. If other solvents are needed, acetonitrile can be quickly removed by evaporation, and the process in other solvent will be studies in the future. The first, in situ method requires that the radiolabeling precursor be more stable than TsF (e.g., tosylate or quaternary ammonium as leaving groups), but when precursors are less stable (e.g., mesylate or triflate), the pre-heating method is required. The lyophilized complex of K_2_CO_3_/K_222_ (1:2) is a white solid that can be easily weighed or prepared as a stock solution. Therefore, as low as 1 mg complex (equivalent to 0.15 mg K_2_CO_3_) can be used to convert [^18^F]TsF to [^18^F]fluoride efficiently (Supplementary Tables S6 and S7). This allows the use of small amounts of precursor under well-controlled conditions, and this is especially useful for base-sensitive precursors or precursors available in only limited amounts.

There are several side reactions that can occur during the nucleophilic substitution reaction, as shown in Fig. 1. The side reactions not only consume precursors but also can generate acids and water, which can quickly reduce radiolabeling efficiency and degrade the labeled product. According to literature^16–18^, [^18^F]fluoride, generated via chemical conversion, usually afforded high RCCs in nucleophilic substitution radiolabeling.

Elimination reactions are one of the major side reactions (Fig. 1). The formation of the eliminated product (vinyl azide)^28^ in a model reaction was studied to explore the micro-environment under different drying conditions (Supplementary Table S8). Much more vinyl azide (a fourfold increase) was observed with azeotropic drying with or without the QMA treatment than those without azeotropic drying. The QMA treatment resulted in even more formation of vinyl azide. Preheating a solution of K_2_CO_3_/K_222_ in acetonitrile, a process to convert [^18^F]TsF to [^18^F]fluoride, resulted in much less vinyl azide. This experiment suggests that the micro-environment is different in radiolabeling reactions using azeotropic drying versus water-free processed fluoride.

Since chloride ions were formed during the process of producing [^18^F]TsF, the presence of chloride ions may interfere with the radiolabeling reaction or the purification of the final product from the chlorine-substituted derivative. Therefore, the chloride content from different processes was studied using a model reaction by detecting the chlorine-substituted derivative formed (Supplementary Table S9). A minimum amount of chlorinated-derivative was observed from the processing of TsF/TsCl, indicating that chloride was trapped efficiently by the resin. In sharp contrast, the commercially available and radiolabeling-ready cartridges (30-PS-HCO_3_ and QMA) generated a more significant amount of chlorine derivative.

### Proof of concept: radiofluorination using ^18^F-labeled sulfonyl fluorides as the [^18^F]fluoride source

**Figure 4:**
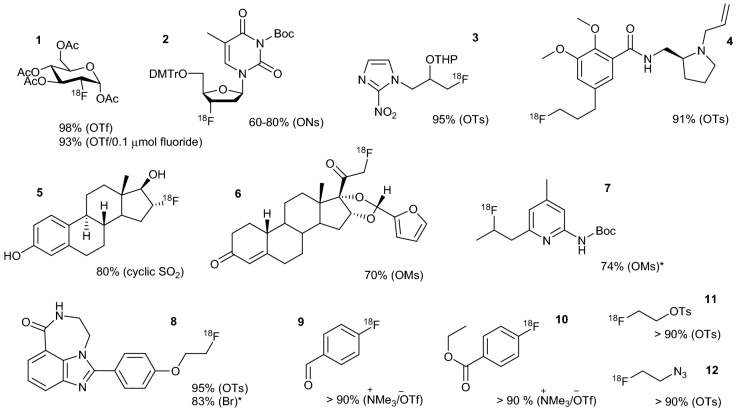
Exploration of nucleophilic radiofluorination using [^18^F]TsF and other sulfonyl fluorides (indicated by *). Radiochemical conversion (RCC) was determined by radio-TLC and the leaving group is indicated in the parentheses. The reaction details of each compound are presented in supplementary Table S16.

The summary of radiolabeling tests with a variety of precursors using [^18^F]TsF, mainly produced by direct manual elution (see supplementary information), as the [^18^F]fluoride source is presented in Fig. 4 and supplementary Table S16. The synthesis of [^18^F]FDG (as the intermediate) afforded almost quantitative RCC (98%) with nearly no radioactivity left in the reaction vessel, in contrast to that up to 20% radioactivity may typically be lost to the reaction vessel using the azeotropic drying procedure with K_2_CO_3_/K_222_. The carrier-added radiosynthesis of [^18^F]FDG, to simulate synthesis using a large amount of radioactivity (0.1 µmol fluoride versus 52 µmol [^18^F]FDG precursor), still gave 93% RCC. A small amount of the other precursors were used to test the radiolabeling but still afforded high RCCs, especially for base-sensitive precursors/products (e.g., [^18^F]FLT, [^18^F]FFNP and [^18^F]FNOS^29^). The use of small amounts of precursors are beneficial for the preparation of ^18^F-labeled radiotracers in many aspects. The radiolabeling reactions are mild, consistent with the differing micro-environments discussed above. The [^18^F]fluoride generated from [^18^F]TsF and other ^18^F-labeled sulfonyl fluorides appear to be more reactive, resulting in high RCCs even with bromide as the leaving group.

### Preparation of ^18^F-labeled radiopharmaceuticals using [^18^F]TsF as the [^18^F]fluoride source

With [^18^F]TsF generated by the peristaltic pump method (Fig. 2), we have used [^18^F]TsF as the [^18^F]fluoride source in the preparation of ^18^F-labeled radiotracers for pre-clinical research (Table 1). A typical preparation started with 1 GBq [^18^F]TsF in 0.5–1 mL acetonitrile, 1–2 mg precursors and 0.5–1 equivalent K_2_CO_3_/K_222_. The radiochemical yields (RCY) after HPLC purification were high. For example, we achieved over 90% RCY for the synthesis of [^18^F]FFNP using a triflate precursor and up to 80% RCY for the synthesis [^18^F]FDHT in a one-pot reaction using only 1 mg precursor. Because of its low molar absorptivity, the A_m_ of [^18^F]FDHT was under the detection limit by HPLC. By contrast, because of its high molar absorptivity, the A_m_ of [^18^F]TsF could readily be measured to give us some information about the A_m_ of [^18^F]FHDT; this is a valuable feature of using [^18^F]TsF for radiolabeling. The molar activity of the synthesized radiotracers was up to 370 GBq/µmol, indicating that little [^19^F]fluoride was introduced during the processing of [^18^F]TsF.
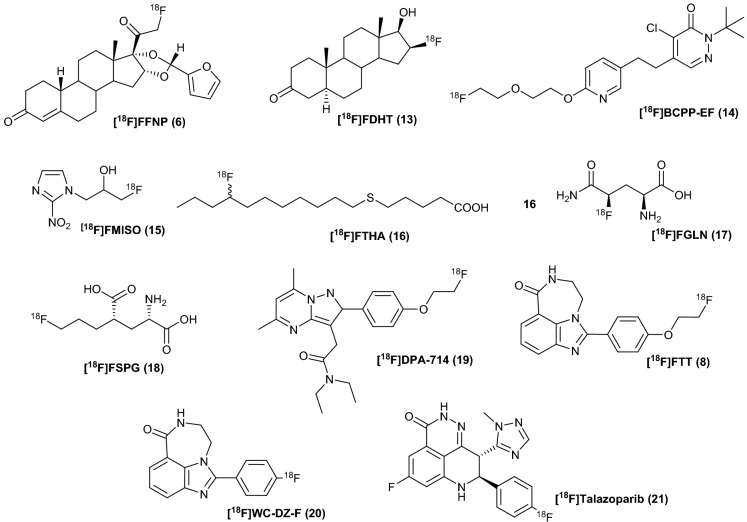

**Table 1:** Radiosynthesis of ^18^F-labeled radiopharmaceuticals using [^18^F]TsF as a [^18^F]fluoride source.

Name	Precursor (mg)	K_2_CO_3_/K_222_ (mg)	RCY^a^ (%)	A_m_ (GBq/µmol)
[^18^F]FFNP (**6**)	2 (OTf)	2.2–3	84.4 ± 8.7 (n = 5)	40.4–91.9
[^18^F]FDHT (**13**)	0.9–1.9	1.7–2.4	76.7 ± 2.2 (n = 4)	38.0–490^b^
[^18^F]BCPP-EF (**14**)^31^	2.5	3	39.7 ± 7.4 (n = 2)	46.6–125
[^18^F]FMISO (**15**)	2	4.3	71 (n = 1)	47.96
[^18^F]FTHA (**16**)	2	2.5	44.9 (n = 1)	/
[^18^F]FGLN (**17**)^33^	1.8–2.2	1.5–1.6	37.2 ± 11.2 (n = 8)	/
[^18^F]FSPG (**18**)	2	1.7–2.4	70.7 ± 7.3 (n = 8)	13.0–407
[^18^F]DPA-714 (**19**)^32^	1	1.8	74.4 (n = 1)	108
[^18^F]FTT (**8**)	0.6–1.2	1.6–3	66.5 ± 8.4 (n = 13)	36.4–292
[^18^F]WC-DZ-F (**20**)^c^	–	–	46.8 ± 10.4 (n = 12)	26.2–372
[^18^F]Talazoparib (**21**)^c^	–	–	56 ± 9.9 (n = 2)^d^	28.1

a. Radiochemical yield (RCY) was the isolated yield after HPLC purification (decayed corrected) starting from [^18^F]TsF; b. Molar activity of [^18^F]TsF; c. The radiosynthesis started from [^18^F]fluorobenzaldehyde [RCC(Radio-TLC): 97%]; d. RCY was reported as the chiral mixture.

As shown in Table 1, the radiolabeling reaction is reliable and reproducible, and the radiolabeling procedure was straightforward for stable precursors. For example, the radiosynthesis of [^18^F]FTT^30^ was readily achieved by adding [^18^F]TsF in acetonitrile to a reaction vessel containing the precursor and K_2_CO_3_/K_222_ and heating for 10 min before dilution for HPLC purification. The total synthesis time could be < 30 min using [^18^F]TsF. Since the precursor is not soluble in acetonitrile at room temperature, this protocol is not an option for conventional radiolabeling using a synthesis module. The use of acetonitrile as the reaction solvent is beneficial for the HPLC purification of [^18^F]FTT, which is very polar. The preparation of less-commonly-used PET radiotracers (e.g., [^18^F]BCPP-EF^31^ and [^18^F]DPA-714^32^) and PET radiotracers with a limited quantity of precursors (e.g., [^18^F]FGLN^33^ 2 mg vs. 10 mg^34^) for research projects is also straightforward, simply by manipulating the amount of precursors and K_2_CO_3_/K_222_ in the radiolabeling reactions without many optimization studies. Less precursors also allowed easy manipulation of radiolabeling reactions and better purification of the final products by HPLC. The preparation of ^18^F-labeled synthons in acetonitrile in high RCCs (e.g., 4-[^18^F]fluorobenzaldehyde ([^18^F]FBAL), 97% RCC) using [^18^F]TsF allows multiple-step radiosynthesis to be carried out with ease. [^18^F]WC-DZ-F^35^ and [^18^F]Talazoparib (as chiral mixture)^36^ were radiosynthesized starting from [^18^F]FBAL in multiple steps in about 50% RCY, enabling the evaluation of these tracers without the need for the development of new precursors and optimization of radiolabeling conditions.

## Discussion

The majority of reported protocols to avoid the azeotropic drying step are based on a trapping-and-elution strategy on an ion exchange resin, including eluting with a solvent containing a small amount of water and followed by direct radiolabeling in the aqueous solution^7^, eluting with non-basic salts (e.g., Bu_4_NOTs^11^) in an alcoholic solution (e.g., methanol or ethanol) and followed by removing the alcoholic solution under a flow of inert gas before radiolabeling reactions. These strategies are simple and compatible with current automated synthesis modules but have drawbacks. As we have demonstrated in the radiolabeling of [^18^F]FDG in the presence of water, the inclusion of water would unavoidably reduce the reactivity of [^18^F]fluoride (thus lowered RCCs) under the conventional heating (see supplementary information). The variety of reagents used for the treatment of ion-exchange resin and as the eluting reagents are not always available in high purities, therefore, there is the attendant risk that [^19^F]fluoride would be introduced into the radiolabeling step, resulting in low A_m_ when a small amount of radioactivity was used^10^. Besides water, methanol and ethanol are commonly used as co-eluting solvents. However, as demonstrated in the control radiolabeling reactions (see supplementary Figure. S34), the residual methanol or ethanol may react with the labeling precursors to form the corresponding methoxy and ethoxy derivatives, respectively, resulting in low “observed” A_m_ due to the frequent coelution of the methoxy derivative with the ^18^F-labeled product and low effective A_m_ if the ethoxy derivative is not separated by HPLC. The eluting step also introduces counter ions from the resin and eluting salts themselves, which might impact each reaction in different ways.

The current standard practice for preparing ^18^F radiotracers is to use a large amount of radioactivity, and thus the above drawbacks may not be an issue. However, this practice is costly, limits the daily multiple use of a hot cell and a synthesis module, and is not ideal for dose-on-demand synthesis^37^ and for the adoption of new technology (microfluidics, etc.)^38^ for radiolabeling. In addition, the development of novel PET imaging technology and enhanced imaging processing of low-dose scans suggest that in the future, only a smaller amount of radioactivity will be required to obtain a high quality image than from current, conventional PET scanners; this will begin to make high activity level radiolabeling less desireable^39^. As demonstrated above, the use of [^18^F]TsF as the [^18^F]fluoride source provides a solution for the efficient and convenient preparation of ^18^F-labeled tracers on a reduced scale that is well matched to the future needs of PET imaging. The radiolabeling using [^18^F]TsF is straightforward and affords high RCYs, perfect for miniaturizing radiolabeling reactions and radiolabeling devices. The processing of [^18^F]TsF introduces a minimum amount of fluoride, ideal for small-scale radiolabeling while maintaining high A_m_. In addition, [^18^F]TsF is storable in acetonitrile, which, at least within the limits of the half-life of fluorine-18, is ideal for dose-on-demand radiosynthesis. Therefore, the use of [^18^F]TsF as the ^18^F source for ^18^F radiolabeling is well suited to meet the emerging needs in the preparation of ^18^F-labeled PET radiotracers.

Unlike conventional radiofluorination, radiolabeling using [^18^F]TsF can be carried out in a well-controlled manner for method development and trouble shooting. For example, we have used [^18^F]TsF to study the effect of water content on the radiosynthesis of FDG, the effect of additives (KOTf and KOTf/K_222_) on nucleophilic substitution radiolabeling and the one-pot/two-step radiolabeling strategy (see Supplementary Data). Recently, we have used [^18^F]TsF to explore the copper-mediated radiofluorination (CMRF) of aryl boronic acid/ester precursors^40^. Preliminary studies also indicate that [^18^F]TsF is better than [^18^F]TfF (higher RCY, because of reduced loss to the reaction vessel and lower introduction of [^19^F]fluoride ion, etc.). (See Supplementary Data). The generation of [^18^F]TsF is an application of continuous flow chemistry, which is a process that can be easily integrated into microfluidic devices for radiolabeling^41^. Therefore, there is still much more for the community to explore with [^18^F]TsF.

## Conclusion

We have demonstrated that [^18^F]TsF is a versatile [^18^F]fluoride source for radiofluorination without the need for an azeotropic drying step and have developed a continuous flow solid-phase strategy to generate [^18^F]TsF in a short time, in high radiochemical yields (RCYs) and purities, at high molar activity (A_m_) with a minimum amount of [^19^F]fluoride introduced during the process and in a form that is ready for radiofluorination. The [^18^F]TsF that is generated shows suitable features for radiofluorination, allowing preparation of ^18^F-labeled PET radiotracers in high RCY, high A_m_ and high reproducibility, exploration of radiofluorination reactions and potential applications in novel technologies. In conclusion, [^18^F]TsF is a promising and versatile [^18^F]fluoride source for the preparation of ^18^F-labeled PET radiotracers and has great potential in the application of novel technologies and concepts (e.g., dose-on-demand and microfluidic radiosynthesis) for the future needs, benefiting the PET imaging society, now and in the future.

## Methods

### General information

All chemicals were obtained from standard commercial sources and used without further purification. No-carrier-added [^18^F]fluoride was produced by an ^18^O(p, n)^18^F reaction through proton irradiation of ^18^O-enriched water (95%) using an RDS111 cyclotron or an ACSI TR-19 cyclotron in the cyclotron facility at Washington University in Saint Louis (WUSTL). The radiolabeling precursors and standards are either made in-house or from commercial sources. The peristaltic pump and its accessories were purchased from Cole-Parmer. Cautions: All processing of radioactive materials should be carried out in a fume hood certified for radioactive work.

### Generation of [^18^F]TsF using a peristaltic pump

The procedure was carried out in a fume hood with proper protection from radiation exposure.

#### Step 1.

 Preparation: The peristaltic pump and corresponding Teflon tube were cleaned up by rinsing with acetonitrile (5 mL) and water (5 mL) at 3 mL/min, followed by attaching a cartridge (30-Ps-HCO_3_/46 mg or MP-1 M-HCO_3_/30 mg) connected with a short needle (1 inch) to the eluting end of the tube and the loading end is attached with a long needle;

#### Step 2.

 Loading and drying: [^18^F]Fluoride (up to 2 GBq) in [^18^O]water (100–500 µL), delivered from the cyclotron facility in a 1-mL syringe, was transferred to a 1.5 mL polypropylene microcentrifuge tube. In order to completely transfer the radioactivity, the syringe was rinsed with water (1 mL), which was also collected in the vial. After the long needle was inserted into the tube, the pump was turned on. After 30 s, water (1 mL) was added to rinse the tube, and after further 60 s, the long needle was inserted into a tube containing acetonitrile (5 mL) for drying;

#### Step 3.

 Generation of [^18^F]TsF: Three 1.5 mL microcentrifuge tubes were prepared as follows: Tube #1 was loaded with a solution of TsCl (1 mg) and TsOH·H_2_O (0.25 mg) in acetonitrile (0.3–0.5 mL); Tube #2 was loaded with acetonitrile (0.3–0.7 mL) for rinsing; Tube #3 was loaded with acetonitrile (1–1.5 mL) for final rinsing. After the long and short needles were inserted into Tube #1, the pump was turned on to circulate the solution for 3 min at a flow rate of 3 mL/min at room temperature, and then the long needle was inserted into Tube #2 while the short needle remained in Tube #1 to collect the eluted radioactivity. Upon completion of the elution, the long needle was inserted into Tube #3, and the short needle was inserted into an empty tube for collection of the final elution (3–5% of total radioactivity), which can be used to determine the molar activity of [^18^F]TsF or for test labeling. All the eluted radioactivity and the cartridge were measured to determine RCY of [^18^F]TsF.

Another option for collection of [^18^F]TsF in a reaction tube: After the circulation, the pump was turned off briefly, and the short needle was inserted into a reaction tube containing K_2_CO_3_/K_222_ and the labeling precursor. [^18^F]TsF was eluted into the reaction tube, followed by rinsing with acetonitrile by adding acetonitrile to Tube #1. The reaction tube was then used for the reaction.

## Supplementary Information

Supplementary Information.

## Data Availability

All data generated or analyzed during this study are included in this published article and its supplementary information file.
