# Supplementary material for: [18F]Tosyl fluoride as a versatile [18F]fluoride source for the preparation of 18F-labeled radiopharmaceuticals
Source: Sci Rep. 2023 Feb 23;13:3182. doi: 10.1038/s41598-023-30200-2 (PMC9950486; doi:10.1038/s41598-023-30200-2)
Supplement: Supplementary file 1 — Supplementary Information. [file 41598_2023_30200_MOESM1_ESM.pdf]

Supplementary Data

[^{18}F]Tosyl fluoride as a versatile [^{18}F]fluoride source for the preparation of ^{18}F-labeled radiopharmaceuticals

Dong Zhou[1,*], Wenhua Chu[1], Jinbin Xu[1], Sally Schwarz[1], John A. Katzenellenbogen[2]

1. Department of Radiology, School of Medicine, Washington University in Saint Louis, Saint Louis, MO 63110, USA
2. Department of Chemistry, University of Illinois at Urbana-Champaign, IL 61801, USA

General information

All chemicals were obtained from standard commercial sources and used without further purification. No carrier-added [^{18}F]fluoride was produced by an ^{18}O(p, n)^{18}F reaction through proton irradiation of ^{18}O-enriched water (95%) using a RDS111 cyclotron or ACSI TR-19 cyclotron in the cyclotron facility at Washington University in Saint Louis (WUSTL). High-performance liquid chromatography (HPLC) was performed with an ultraviolet detector (UV) and a radiation detector. An Altima C18 250 × 4.6 mm 10 μm analytical column was used with acetonitrile and water with 0.1% TFA as the HPLC mobile phase for the analysis of [^{18}F]TsF and test radiolabeling reactions unless otherwise stated. An Agilent SB-C18 250 × 9.4 mm 5 μm column or a Phenomenex Luna C18 250 × 10 mm 5 μm column was used for purification of ^{18}F-labeled radiopharmaceuticals. Radio-TLC was accomplished using a Bioscan AR-2000 imaging scanner (Bioscan, Inc., Washington, DC) (TLC plate: Silica gel; Solvent: Ethyl acetate or 20% Methanol in dichloromethane). The radiolabeling precursors and standards are either made in-house or from commercial sources. 4-Toluenesulfonyl chloride (TsCl) was purchased from Sigma Aldrich (saint Louis, MO, USA). The peristaltic pump (Masterflex® L/S® Modular Drive and pump head) and its accessories (tube) were purchased from Cole-Parmer (Chicago, IL, USA). Cautions: All processing of radioactive materials should be carried out in a fume hood certified for radioactive work.

Preparation of MP-1M-HCO_3

Into a glass column was loaded AG MP-1M resin (200-400 mesh, chloride form, cat#141-1851, 2 cm × 10 cm). The resin was rinsed at 3 mL/min with 1. Water (200 mL); 2. 1 M $KHCO_3$ solution (600 mL); 3. Water (500 mL); 4. Acetonitrile (200 mL), and then dried under high vacuum to produce free flow resin. The resin was stored at 5 °C. The resin is about 3 meq/gram.

Preparation of potassium carbonate/Kryptofix 222 (K_2CO_3/K_{222})

Potassium carbonate (99.995% purity, 1.347 g, 9.75 mmol) and Kryptofix 222 (3.668 g, 9.74 mmol) were dissolved in 70% water/30% acetonitrile (30 mL), and the solution was loaded into 50 mL polypropylene Centrifuge Tubes (4 ×). After the solution was frozen at -78 °C, it was lyophilized using a Labconco FreeZone 6L freeze dryer (Cold trap at -45 °C and sample at -20 °C) for two days or until no further vacuum drop or loss of sample weight. The dried sample was allowed to warm up to room temperature and transferred to a glass vial. The bulk of the dried sample was stored at 5 °C and the sample ready for use was stored at room temperature.

Note: Drying the sample further under high vacuum at room temperature will result in a sample not completely dissolved in acetonitrile and a solution with yellowish color. According to the weight loss under high vacuum at room temperature, there is about 1.5% water in the dried K_2CO_3/K_{222} as described above.

Manual elution: A typical example

As shown in **Fig. S1**, [^{18}F]fluoride in water (1 mL) was loaded onto a 30-PS-HCO_3 cartridge under reduced pressure and the eluted [^{18}O]water was collected. The cartridge was dried by rinsing with acetonitrile (1 mL) at RT and (2 mL) heated at 80 °C oil bath, and then a solution of TsCl (1 mg) in acetonitrile (0.5 mL), heated in a 80 °C oil bath, was loaded, passed through the cartridge and collected in a tube. The cartridge was then rinsed with acetonitrile (0.5 mL), and the solution was collected in the same tube. The elution efficiency was 93.8% by counting the eluted radioactivity (0.56 GBq/15 mCi) and the radioactivity left on the cartridge and tubing (0.037 GBq/1 mCi). According to radio-HPLC, the eluted solution contains TsCl (70 µg). Radiochemical purity is 100% and molar activity of [^{18}F]TsF is 3393 mCi/µmol at the end of elution.

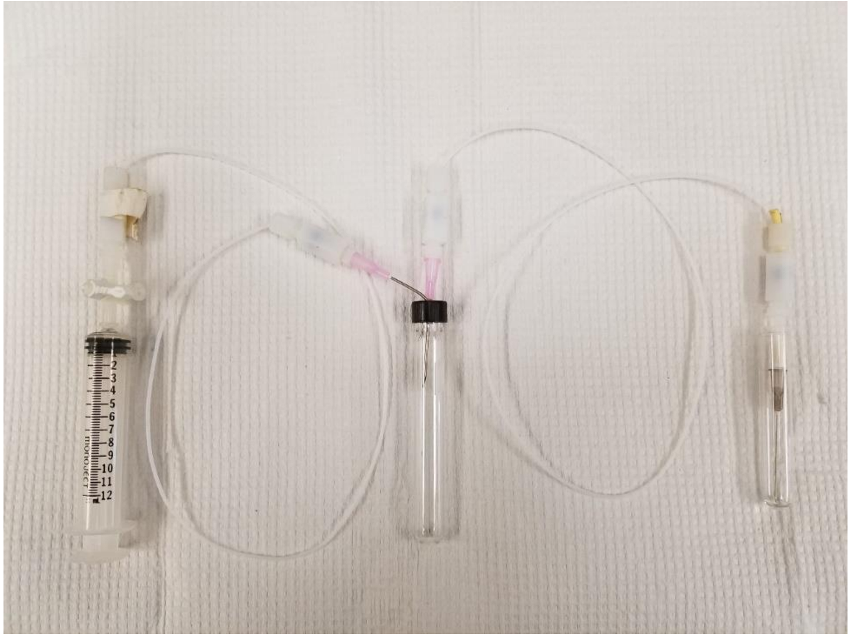

Figure S1. The manual elution setup: A 12 mL syringe (left) was used to provide the reduced pressure for loading. Different tubes (middle) were used to collect water/acetonitrile and the final radioactivity via the cartridge from corresponding tubes (right), containing [^{18}F]fluoride in water, acetonitrile and a solution of TsCl in acetonitrile.

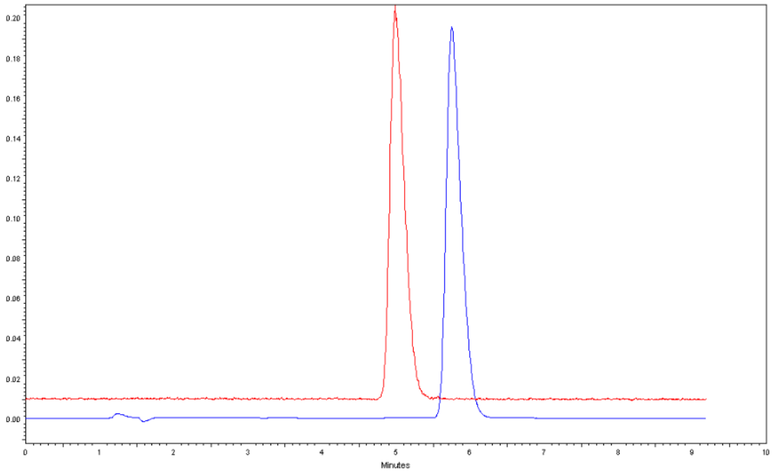

Figure S2. Analytical radio-HPLC of directly eluted [^{18}F]TsF, showing a significant amount of TsCl (Red: UV; Blue: radioactivity). The radioactive peak at 5 min is [^{18}F]TsF and UV peak at 6 min is TsCl (HPLC condition: Altima C18 250 × 4.6 mm, 60% MeCN/40% water/0.1% TFA, 2 mL/min, 228 nm).

Table S1. Formation of [^{18}F]sulfonyl fluoride in aqueous solution

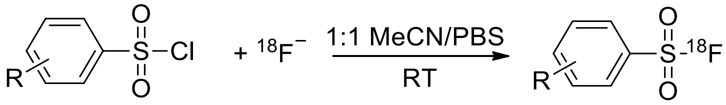

R	RCC (%)[a]				
	5 min	10 min	15 min	30 min	60 min
4-MeO	7.7	14.7	18.6	30.9	46.2
2-Me	16	36.1	53.14	70.6	85.9
3-Me	13	27.6	50.2	59	81.2
4-Me	10.6	19	27.4	50.3	75.3
2-F	24.8	37.5	53.9	81.3	93.6
3-F	39.4	57.8	78.2	94.8	100
4-F	17.2	35.3	48.2	77.7	90.7
2-Cl	24.4	43.5	77.1	91	98.7
3-Cl	37.9	69	70.5	98.9	100
4-Cl	41.9	69.8	82.3	95.1	98.1
4-Br	34.3	56.5	83.6	95.2	99.5
4-I	27.8	57.6	82.2	95.7	99.7
2-CF_3	57.5	71.1	92.2	100	100
3-CF_3	53.8	79.2	90.3	100	100
4-CF_3	70.8	91	96	100	100
CH_3CO	79.5	96.8	99.3	100	100
2-CN	90.6	97.1	98	100	100
4-CN	92.4	97.4	98	98.5	98.1
2-Py	99.2	99.3	99.1	98.9	98.3
4-NO_2	96.7	97	96.5	97.3	98.3
MsOMs	0		0		

Note: a. Typical reaction condition: Into a polypropylene vial were loaded PBS buffer (1×, pH = 7.4, 500 µL), sulfonyl chloride (5.25 µmol) in acetonitrile (500 µL) and [^{18}F]fluoride in water (10 µL). The mixture was vortexed briefly, and radiochemical conversion (RCC) of [^{18}F]fluoride was determined by radio-TLC.

Table S2. Elution of [^{18}F]tosyl fluoride in different solvents

Solvent	TsCl (mg)[a]	Temp	RCC(%)[c]
MeCN	1	80°C	95
MeCN	2 × 1[b]	80°C	96.7
MeCN	1	RT	95
Amyl alcohol	1	80°C	98
THF	1	RT	92.5
1,2-Dichlorobenzene	1	RT	36
EtOH	1	RT	96.7
DMF	1	RT	77
DMF	1	80°C	93.6
DMSO	1	80°C	0

Note: a. A solution of TsCl (1 mg, 5.25 μmol) in the chosen solvent (0.5 mL) was used to elute the radioactivity, followed by rinsing with the chosen solvent (0.5 mL); b. KF (0.29 mg, 4.99 μmol) was added to [^{18}F]fluoride; c. Radiochemical conversion (RCC) was determined by the eluted radioactivity over total radioactivity.

Table S3. Elution of [^{18}F]TsF using different anion exchange resin[a]

Resin[b]	RCC (%)
Chromafix 30-PS-HCO_3	95-97
Bio-Rad MP-1M	90-95
Bio-Rad Bio-Rex 5	91-94
Chromafix PS-OH	91
Phenomenex Strata-X-A	90-95
Waters QMA	49
Waters MAX	93

Note: a. A solution of TsCl (1 mg, 5.25 μmol) in acetonitrile (0.5 mL) was used to elute the radioactivity, followed by rinsing with acetonitrile (0.5 mL); b. Resin (~50 mg) was treated by rinsing sequentially with acetonitrile (5 mL), water (5 mL), 1 M $KHCO_3$ (10 mL) and water (5 mL); c. Radiochemical conversion (RCC) was determined by the eluted radioactivity over total radioactivity.

Table S4. Elution of ^{18}F sulfonyl fluoride with different eluting agents

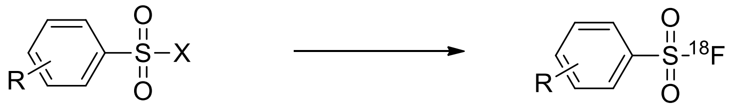

R[a]	X	RCC (%)
4-NO_2	Cl	70-90
2-CN	Cl	60-80
4-Me	OTs	92
2-Cl	Cl	79
4-MeO	Cl	93
2,4,6-trimethylbenzene	Cl	95.3
4-CF3	Cl	86
2-Pyridinesulfonyl chloride		80
MsOMs		58

Note: a. Direct elution using 1 mg sulfonyl chloride in acetonitrile.

Table S5. Effect of flow rate[a]

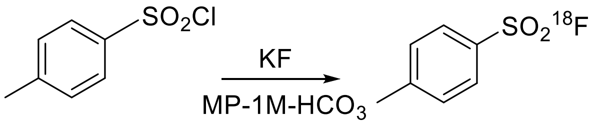

Entry	Flow rate (mL/min)	Time (min)	HPLC integration[b]		Note
			TsF	TsCl	
1	2	2	186 ± 2	9 ± 0	
2	3	2	199 ± 4	20 ± 0	
3	3	3	201 ± 4	0	
4	3	4	197 ± 1	0	
5	4.5	2	196 ± 1	10	
6	6	1.5	203 ± 3	33 ± 1	
7	3 (30-PS-HCO_3)	3	202 ± 2	0	

Note: a. A solution of KF (5.8 µmol) in water (1 mL) was loaded onto an MP-1M-HCO3 (30 mg) cartridge. The cartridge was rinsed with water (1 mL) and acetonitrile (5 mL), and then a solution of TsCl (1 mg) and TsOH·H_2O (0.25 mg) in acetonitrile (0.5 mL) was circulated through the cartridge for the specified time at the specified flow rate. Finally, the cartridge was rinsed with acetonitrile (2 mL) and all the eluted solution was combined for HPLC analysis (n=2); b. The number is the peak integration/1000.

Table S6. Conversion of TsF to fluoride with K_2CO_3/K_{222}

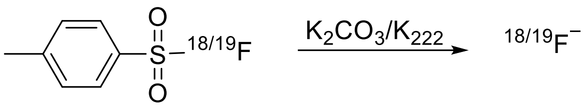

Exp.[a]	1	2	3	4	5	6	7
TsF (μmol)	0.05	0.25	0.5	1	2.5	0.5	0
[^{18}F]TsF (μL)	50	50	50	50	50	50	50
K/K (μmol)[b]	2.81	2.81	2.81	2.81	2.81	1.4	1.4
MeCN (μL)[c]	500	500	500	500	500	500	500
RCC (%)[d]	100	100	100	79.9	61.8	65.8	100

Note: a. Reaction condition: 108 °C/3 min; b. K/K: K_2CO_3/K_{222} (M.W. 891); c. The total volume of acetonitrile; d. Radiochemical conversion (RCC) was determined by radio-TLC.

Table S7. Conversion of TsF/[^{18}F]TsF to [^{18}F]fluoride

Exp.[a]	1	2	3	4	5
TsF (μmol)	1.07	0.535	0.214	0.107	0
[^{18}F]TsF (μL)	50	50	50	50	50
K/K (μmol)[b]	1.1	1.1	1.1	1.1	1.1
MeCN (μL)[c]	500	500	500	500	500
RCC (%)[d]	66	86	97	97	100

Note: a. Reaction condition: 105 °C/7 min; b. K/K: K_2CO_3/K_{222} (M. W. 891); c. Total volume of acetonitrile; d. Radiochemical conversion (RCC) was determined by radio-TLC.

Table S8. Micro-environment study: elimination reaction under different conditions

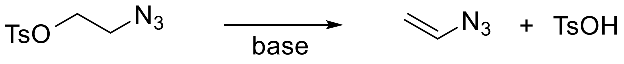

Entry	Processing method	Additive	HPLC integration[e] (Vinyl azide)	Note
1	None	/	93	
2	None	Water (2 μL)	70,84	n = 2
3	None	MeOH (1 μL)	160	
4	Pre-heating[b]	/	21	
5	azeotropic drying[c]	/	354	
6	azeotropic drying[c]	Water (2 μL)	345	
7	QMA[d]/azeotropic drying[c]	/	420	

Note: a. Reaction condition: Precursor (2 μL), K_2CO_2/K_{222} (5 mg), acetonitrile (0.5 mL), 80 °C, 8 min; b. The solution of K_2CO_2/K_{222} was heated at 100 °C for 10 min before the addition of precursor; c. A solution of K_2CO_2/K_{222} in 1:1 acetonitrile/water (0.5 mL) was dried under a standard

drying protocol (105 °C, N_2, 3 × 1 mL acetonitrile); d. A solution of K_2CO_2/K_{222} in 1:1 acetonitrile/water (0.5 mL) passed a Waters QMA-CO_3 (46 mg) cartridge; e. The number is the integration/1000.

Table S9. Measurement of chloride in the process of fluoride via a model reaction

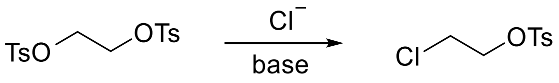

Entry	Processing	HPLC peak integration[a]		Note
		Before heating	After heating	
1	Blank control	78	153	
2	TsCl (0.1 mg)	/	653	
3	TSCl after 30-PS-HCO3 elution	68	154	TsCl (0.5 µg)
4	30-PS-HCO_3	/	1732	
5	30-PS-CO_3	58	152	Treated with K_2CO_3[b]
6	QMA-OH 130 mg	86	4226	
7	QMA-CO_3 130 mg	67	169	Treated with K_2CO_3[b]
8	QMA-CO_3 46 mg (Rotem)	89	1008	Elution #1
9		92	179	Elution #2
10		89	156	Elution #3
11	QMA-CO_3 46 mg (Waters)	/	444	

Note: a. Reaction condition: Precursor (3.2 mg), K_2CO_3/K_{222} (1:2 5 mg), acetonitrile (0.5 mL), 105 °C, 8 min. Cartridge was eluted with K_2CO_3/K_{222} (1:2 5 mg) in acetonitrile (300 µL) and water (200 µL), and the eluted solution was dried under a standard drying protocol (105 °C, N_2, 3 × 1 mL acetonitrile). The reaction mixture (10 µL) was analyzed by analytical HPLC (Altima C18 250×4.6 mm, 60% acetonitrile/40% water/0.1% TFA, 2 mL/min, 240 nm, retention time = 4.05 min). The precursor contains the chlorine-derivative. The number is the integration/1000; b. The cartridge was pre-treated with 0.5M K_2CO_3 (10 mL) and water (15 mL).

Table S10. Effect of water content on FDG radiosynthesis

Water (µL)	0	1	2.5	5	10
Water (ppm)	0	1000	2500	5000	10000
RCC (%)	93.3	87.5	77.5	67.2	49.7

Note: Reaction: A solution of K_2CO_3/K_{222} (5 mg) in acetonitrile (150 µL) with water (0-10 µL) was added an aliquot of [^{18}F]TsF in acetonitrile (50 µL). After the above mixture was heated at 80 °C for 2 min, FDG precursor (5 mg) in acetonitrile (200 µL) was added and the mixture was heated at 80 °C for 7 min. RCC was determined by radio-TLC.

Table S11. Effect of TfOK/K_{222} on radiofluorination

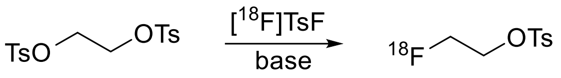

Precursor (mg)	K_2CO_3/K_{222} (mg)	KOTf/K_{222}[a] (mg)	MeCN (µL)	Temp/Time (°/min)	RCC[b] (%)	HPLC profile[c]	Note
2	5	0	500	105/10	92.2	same	
2	5	0.5	500	105/10	91.2	same	
2	5	1	500	105/10	92.8	same	
2	5	2	500	105/10	91.0	same	

Note: a. KOTf/K_{222} was prepared by dissolving 1:1 of KOTf and K_{222} in acetonitrile; b. Radiochemical conversion (RCC) was determined by radio-TLC; b. UV and radioactivity profiles of reaction mixture.

Table S12. Effect of KOTf/K_{222} on radiofluorination

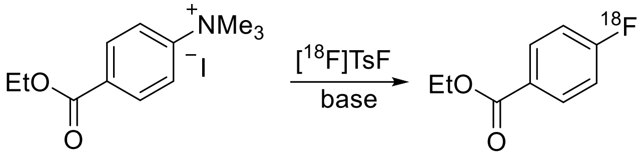

Precursor (mg)	K_2CO_3/K_{222} (mg)	KOTf/K_{222} (mg)	MeCN (µL)	Temp/Time (°/min)	RCC[a] (%)	HPLC profile[b]	Note
2	2.5	0	500	105/10	83.9	same	
2	2.5	0.5	500	105/10	83.5	same	
2	2.5	1	500	105/10	81.1	same	
2	2.5	2	500	105/10	87.0	same	

Note: a. Radiochemical conversion (RCC) was determined by radio-TLC; b. UV and radioactivity profiles of reaction mixture.

Table S13. Effect of KOTf on radiofluorination

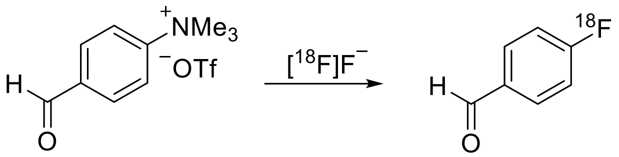

Precursor (mg)	K_2CO_3/K_{222} (mg)	KOTf (mg)	MeCN (µL)	Temp/Time (°/min)	RCC[a] (%)
2	2.5	0	400	110/6	95
2	2.5	2.2	400	110/6	3

Note: a. Radiochemical conversion (RCC) was determined by radio-TLC.

Table S14. One-pot two-step model labeling reaction

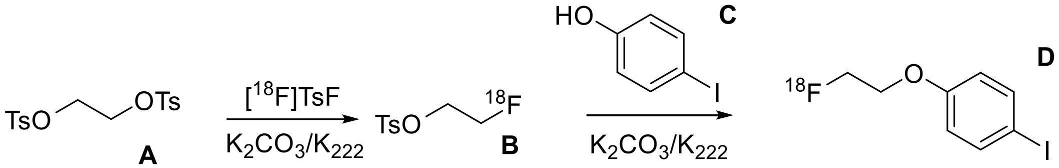

**A** (mg)	K_2CO_3/K_{222} (mg)	[18F]TsF/MeCN (µL/ µL)	RCC (%)[a,b]	**C/**K_2CO_3/K_{222} (mg)	RCC (%)[a,c]	Note
0.5	2.5	100/500	54	0.6/0	35	12% as **B**
0.5	1.25	100/500	78	0.6/2.5	72	
1.0	2.5	100/500	ND	0.6/2.5	57.5	

Note: 1. Radiochemical conversion (RCC) was determined by radio-HPLC; b. Reaction condition: 105 °C/7 min; c. Reaction condition: 105 °C/10 min.

Table S15. Comparation of [^{18}F]Tsf and [^{18}F]TfF

Compound	RCC (%)		Note
	[^{18}F]Tsf	[^{18}F]Tff	
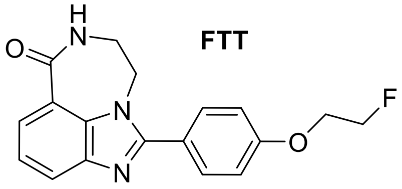 **FTT**	66.5 ± 8.4 (n = 13)	57%	Isolated yields
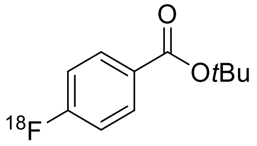	98.5%(7.8%)	96.4%(10.5%)<br>96.4%(14.7%)*	TLC<br>*0.5 µL water added
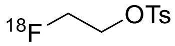	97.6%(10%)	93.4%(20.8%)	HPLC
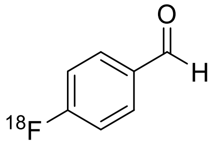	95%(13.3%)	69%(26.7%)	TLC
[^{18}F]fluoride	0.00038 µmol	0.0024 µmol	From processing of sulfonyl fluoride

Note: a. Radiochemical conversion (RCC) was determined by radio-TLC or radio-HPLC of the reaction solution. The percentage of radioactivity left in the reaction vessel over total radioactivity is noted in the parentheses.

General information for test radiolabeling

Method A: A fraction of [^{18}F]TsF elution (50-500 µL) was heated with K_2CO_3/K_{222} at 100-105 °C for 2-5 min, and then a solution of the radiolabeling precursor in acetonitrile was added. The test radiolabeling was carried out under the condition indicated in **Table S16**. Upon completion of the reaction, the mixture was analyzed by radio-TLC for RCC and by radio-HPLC to identify the major radioactive product. The radioactivity left in the reaction vessel was measured to determine the loss of radioactivity to the container.

Method B: The same as described above except that the test radiolabeling was carried out in one pot without the pretreatment step.

Table S16. Radiofluorination using [^{18}F]TsF and other ^{18}F-labeled sulfonyl fluorides-proof of concept[a]

Name	Precursor (mg)	Leaving group	K_2CO_3/K_{222} (mg)	MeCN (mL)	Source of ^{18}F[c]	Condition (°C/min)	RCC[d] (%)	Note for RCC[f]
[^{18}F]FDG (**1**)[a]	5	OTf	5	0.5	([^{18}F]TsF)	80/7	98	3%
[^{18}F]FDG (**1**)[a]	5	OTf	5	1.3	([^{18}F]4ClSF)	80/7	98	5.4%
[^{18}F]FDG (**1**)[a]	25	OTf	27.8	1.3	([^{18}F]TsF)	84/7	93	1.1%
[^{18}F]FLT (**2**)[a]	1.8	ONs	1	0.5	[^{18}F]TsF	105/7	74	25%
[^{18}F]FLT (**2**)[a]	2.1	ONs	2	0.5	[^{18}F]TsF	105/7	73	15%
[^{18}F]FLT (**2**)[a]	6	ONs	5.6	0.7	([^{18}F]TsF)	105/7	76.7	7.6%
[^{18}F]FLT (**2**)[a]	5	ONs	2.5	0.7	([^{18}F]TsF)	105/7	85.9	10%
[^{18}F]FMISO (**3**)[a]	2	OTs	2.5	0.5	[^{18}F]TsF	102/10	95	ND
[^{18}F]Fallypride (**4**)	1	OTs	3	0.6	([^{18}F]TsF)	105/7	91.5	11.2
[^{18}F]FES (**5**)	0.3	Cyclic SO_2	2.2	0.5	[^{18}F]TsF	105/7	80	After hydrolysis[g]
[^{18}F]FFNP (**6**)	1.8	OMs	2.5	0.6	([^{18}F]TsF)	65/7	70.4	21%
[^{18}F]FNOS (**7**)[a]	4.1	OMs	3.6	0.5	([^{18}F]2CNSF)	100/7	74.2	ND
[^{18}F]FTT (**8**)	1.3	OTs	4.1	0.5	([^{18}F]TsF)	105/10	95.7	17.3
[^{18}F]FTT (**8**)	1.8	Br	2.9	0.5	[^{18}F]NsF	105/10	83.4	18.3
[^{18}F]FBAL(**9**)	3.2	$^+NMe_3I$	6 (KH/K)[b]	0.5	[^{18}F]TsF	105/9	94	14.5%
[^{18}F]FBAL(**9**)	4	$^+NMe_3OTf$	5.7	0.5	[^{18}F]TsF	102/8	97	3.3%
[^{18}F]FBA (**10**)	2.8	$^+NMe_3OTf$	5.1 (KH/K)[b]	0.5	[^{18}F]TsF	105/10	93	11%
[^{18}F]FBA (**10**)	3	$^+NMe_3OTf$	7.2	1	[^{18}F]TsF	102/10	92.1[e]	8% as CH_3F[h]
[^{18}F]FETs (**11**)	3.8	OTs	6.1	0.5	[^{18}F]TsF	105/7	97.4	ND
[^{18}F]FETs (**11**)	3.2	OTs	5	0.5	[^{18}F]TsF	105/10	93.1	2%
[^{18}F]FETs (**11**)	2.8	OTs	2.2	0.5	[^{18}F]TsF	105/7	97.8	ND
[^{18}F]FETs (**11**)	1	OTs	2	0.6	[^{18}F]4ClSF	105/9	96.8	ND
[^{18}F]FEAz (**12**)	2.2	OTs	4.9	0.5	[^{18}F]TsF	80/8	95	ND
[^{18}F]FEAz (**12**)	2.4	OTs	5	0.7	[^{18}F]TsF	80/10	99	ND

Note: a. As the intermediate of indicated compounds; b. KH/K: Potassium bicarbonate/K_{222} (1 : 1); c. Parentheses indicate that the sulfonyl fluoride was converted to [^{18}F]fluoride before radiolabeling reactions. [^{18}F]4ClSF: 4-Chlorobenzenesulfonyl [^{18}F]fluoride, [^{18}F]4CNSF: 2-cyanobenzenesulfonyl [^{18}F]fluoride, [^{18}F]NsF: 4-nitrobenzenesulfonyl [^{18}F]fluoride; d. Radiochemical conversion (RCC) was determined by radio-TLC analysis of radiolabeling reaction; e. RCC after hydrolysis with1 N NaOH at 102 °C/5 min; f. The value is the loss of radioactivity to the glass reaction tubes over total radioactivity. ND: not determined. g. 1M H_2SO_4, 105 °C/10 min; h. Volatile radioactivity loss (presumably as [^{18}F]fluoromethane).

Radiosynthesis of FDG (carrier-added)

[^{18}F]TsF was processed from carrier-added [^{18}F]fluoride (0.1 μmol) in 94% RCC using a peristaltic pump (3 mL/min).

[^{18}F]Fluoride (~0.1 GBq) in water (0.5 mL) with KF (0.1 μmol) was loaded onto an MP-1M-HCO_3 (30 mg) cartridge. The cartridge was rinsed with water (1 mL) and acetonitrile (5 mL). A solution of TsCl (1 mg) and TsOH·H_2O (0.25 mg) in acetonitrile (0.4 mL) was circulated through the cartridge for 3 min, followed by rinsing with acetonitrile (0.6 mL) to generate [^{18}F]TsF (0.079 GBq/2.13 mCi) in acetonitrile (1 mL). The cartridge was further rinsed with acetonitrile (1 mL), resulting in 0.0013 GBq (0.034 mCi) radioactivity eluted and 0.0037 Gbq (0.10 mCi) left in the cartridge.

Radiosynthesis of [^{18}F]FDG-intermediate

The solution of [^{18}F]TsF was added to K_2CO_3/K_{222} (27.8 mg, 31.2 μmol), and then heated at 100 °C for 3 min. A solution of FDG precursor (25 mg, 52 μmol) in acetonitrile (0.3 mL) was added, and the mixture was heated at 84 °C for 7 min. RCC of the reaction is 93% according to radio-TLC analysis of the reaction mixture (Silica gel/ethyl acetate) and 95% according to radio-HPLC. 1.1% of total radioactivity was lost to the reaction tube.

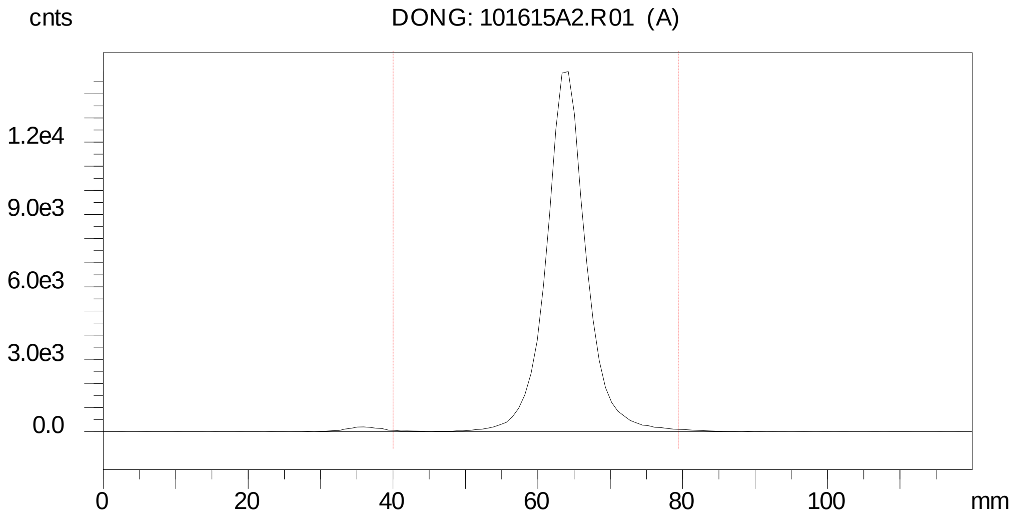

(**a**)

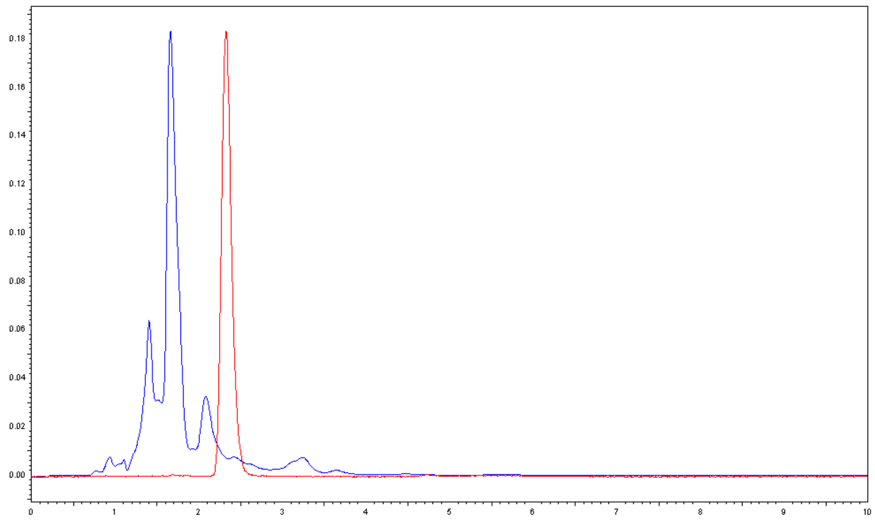

(**b**)

Figure S3. Radio-TLC (**a**) and radio-HPLC (**b**) of [^{18}F]FDG-intermediate (**1**) radiolabeling reaction. (HPLC condition: Altima C18 250 × 4.6 mm, 60% MeCN/40% water/0.1% TFA, 2 mL/min, 254 nm) UV: Blue; Radioactivity: Red.

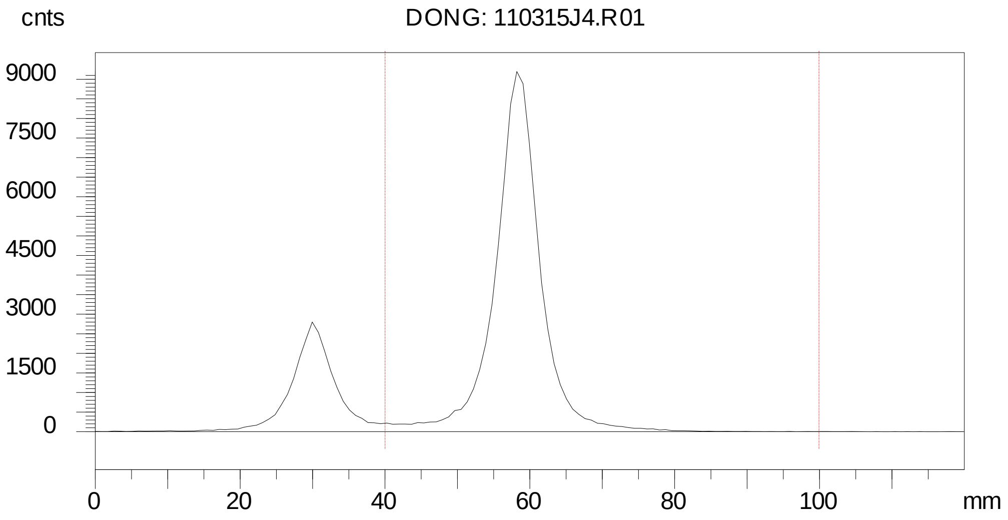

(**a**)

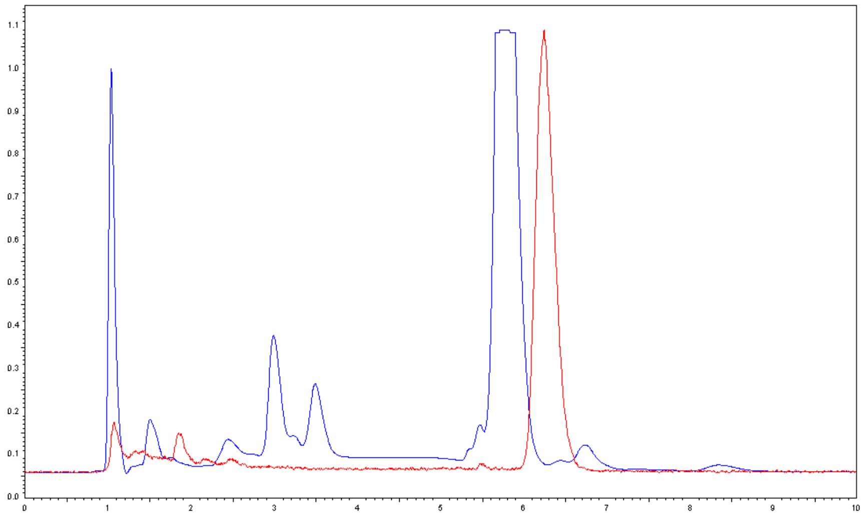

(b)

Figure S4. Radio-TLC (**a**) and radio-HPLC (**b**) of [^{18}F]FLT-intermediate (**2**) radiolabeling reaction (HPLC condition: Altima C18 250 × 4.6 mm, 80% MeCN/20% water/0.1% TFA, 2 mL/min, 254 nm). UV: Blue; Radioactivity: Red.

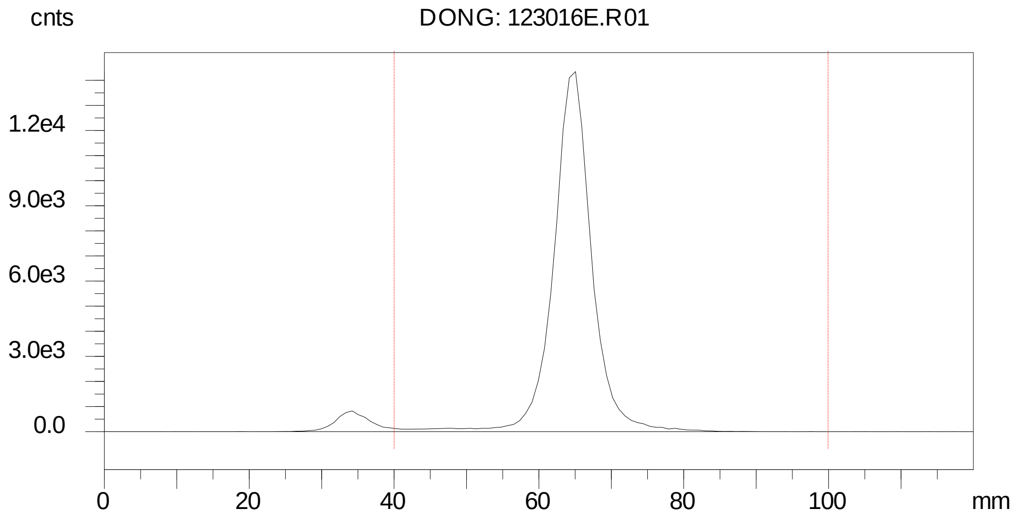

(**a**)

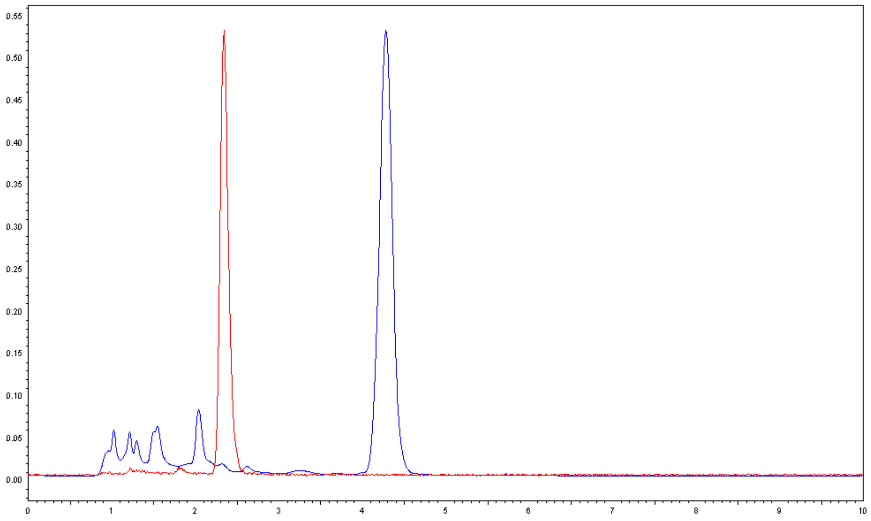

(b)

Figure S5. Radio-TLC (**a**) and radio-HPLC (**b**) of [^{18}F]FMISO (**3**) radiolabeling reaction (HPLC condition: Altima C18 250 × 4.6 mm, 60% MeCN/40% water/0.1% TFA, 2 mL/min, 254 nm). UV: Blue; Radioactivity: Red.

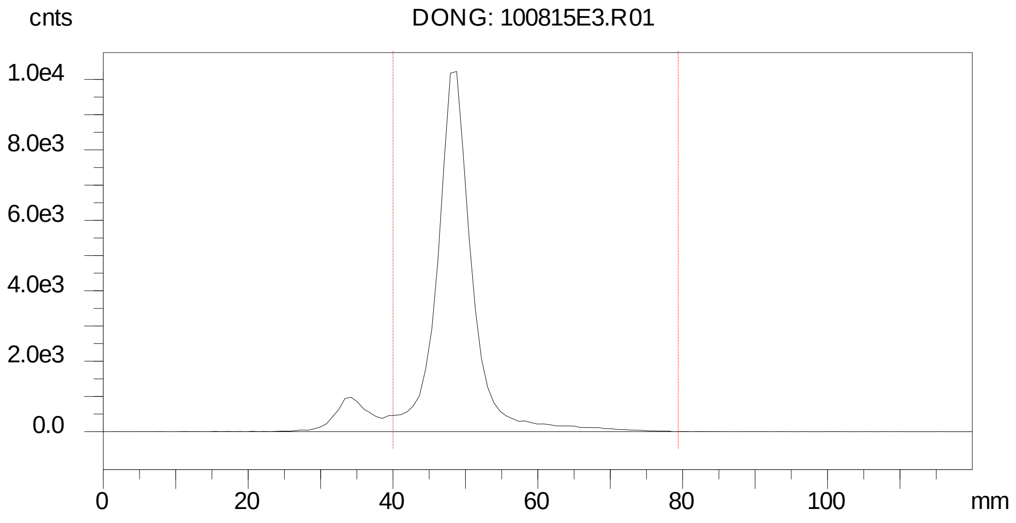

(**a**)

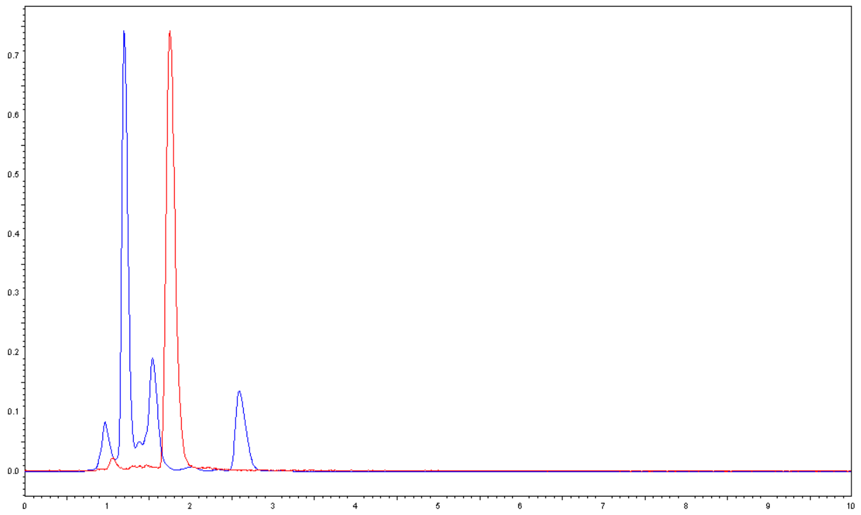

(b)

Figure S6. Radio-TLC (**a**) and radio-HPLC (**b**) of [^{18}F]Fallypride (**4**) radiolabeling reaction (HPLC condition: Altima C18 250 × 4.6 mm, 60% MeCN/40% water/0.1% TFA, 2 mL/min, 254 nm). UV: Blue; Radioactivity: Red.

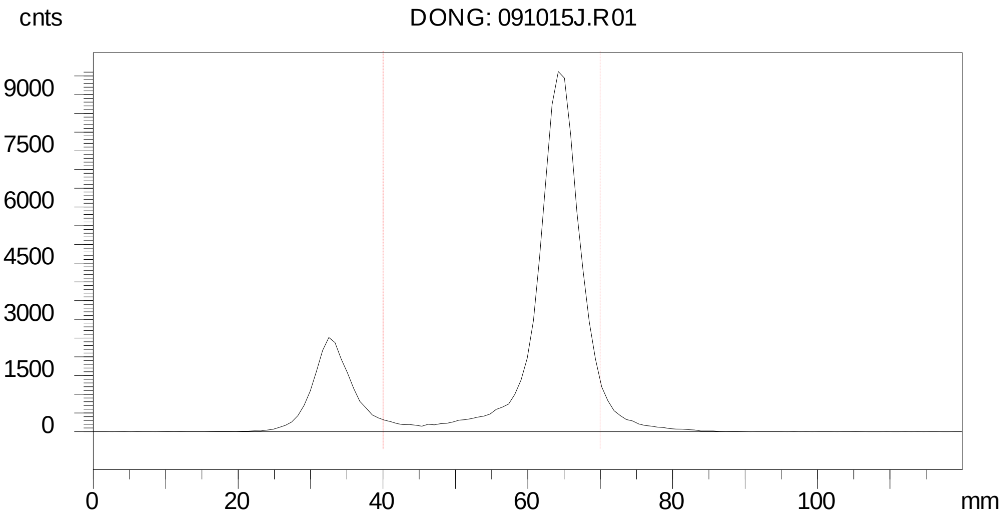

(**a**)

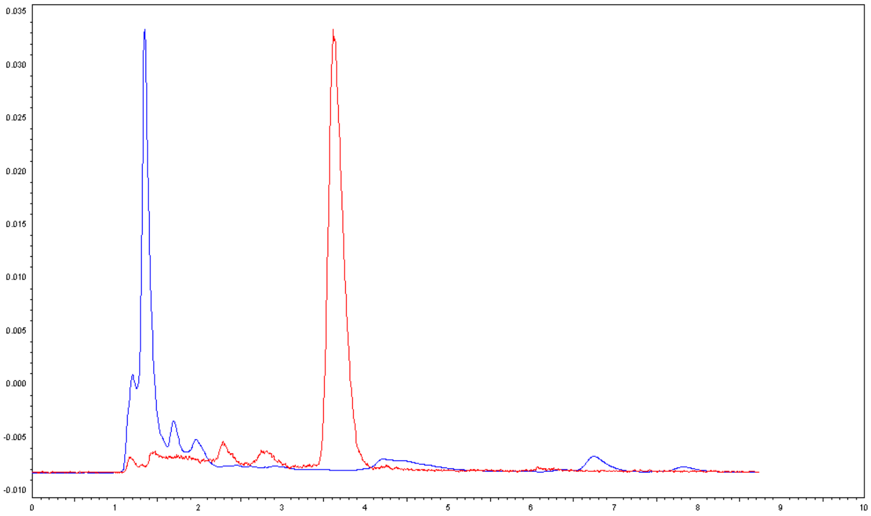

(b)

Figure S7. Radio-TLC (**a**) and radio-HPLC (**b**) of [^{18}F]FES (**5**) radiolabeling reaction (HPLC condition: Altima C18 250 × 4.6 mm, 60% MeCN/40% water/0.1% TFA, 1.5 mL/min, 254 nm). UV: Blue; Radioactivity: Red.

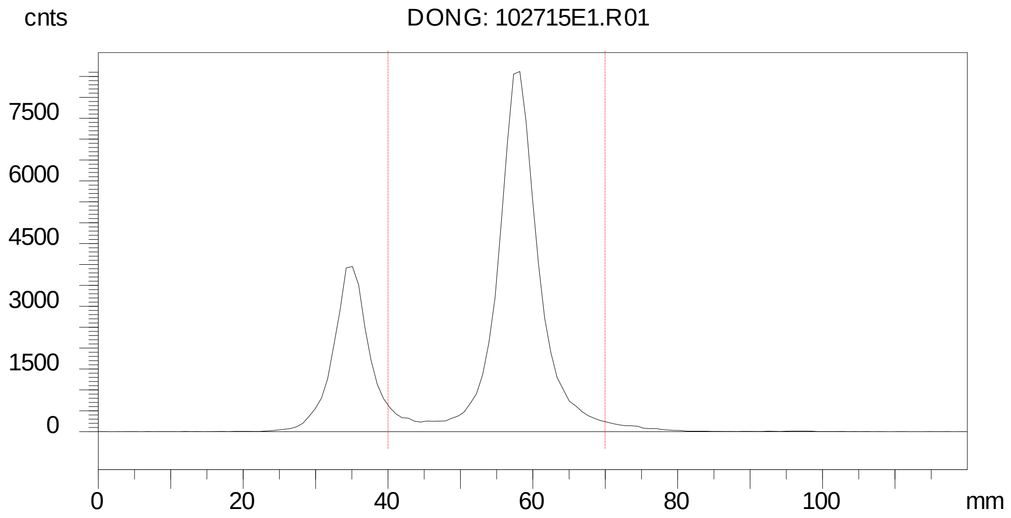

(**a**)

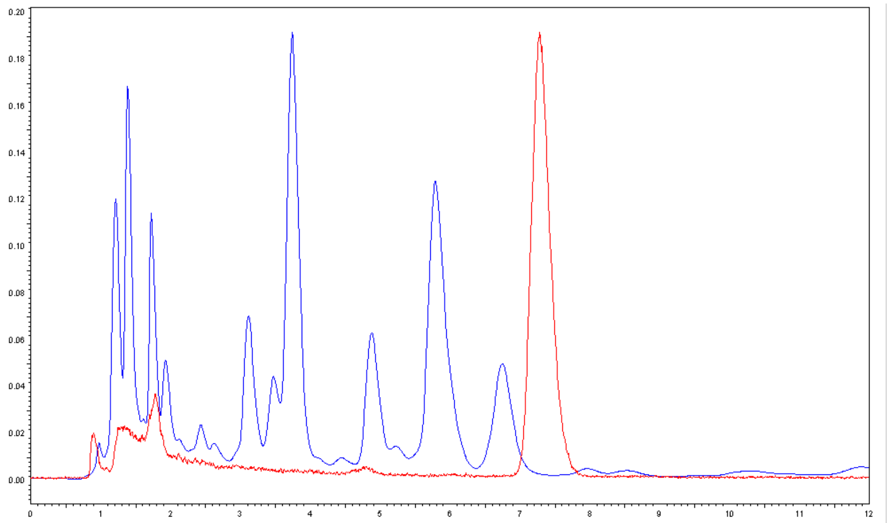

(b)

Figure S8. Radio-TLC (**a**) and radio-HPLC (**b**) of [^{18}F]FFNP (**6**) radiolabeling reaction (HPLC condition: Altima C18 250 × 4.6 mm, 54% MeCN/46% water, 2 mL/min, 254 nm). UV: Blue; Radioactivity: Red.

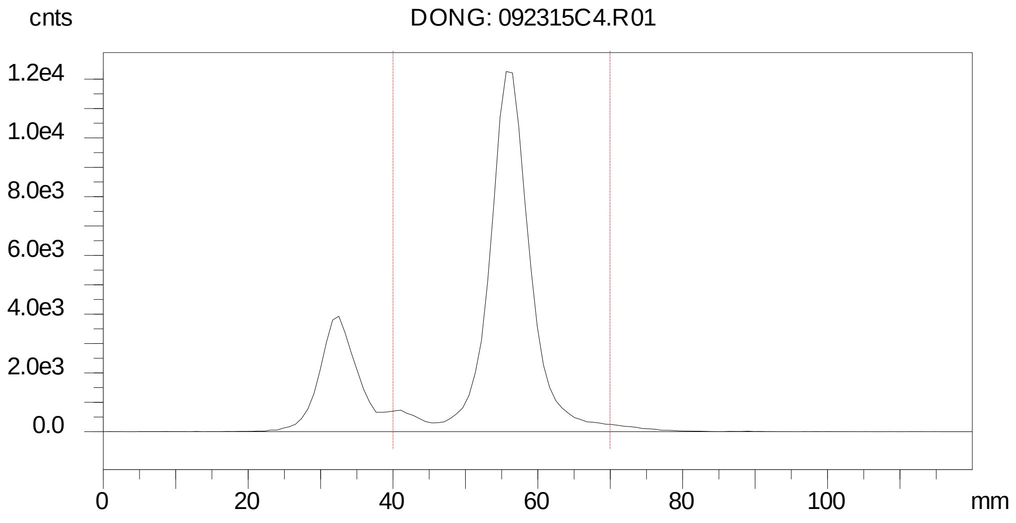

(**a**)

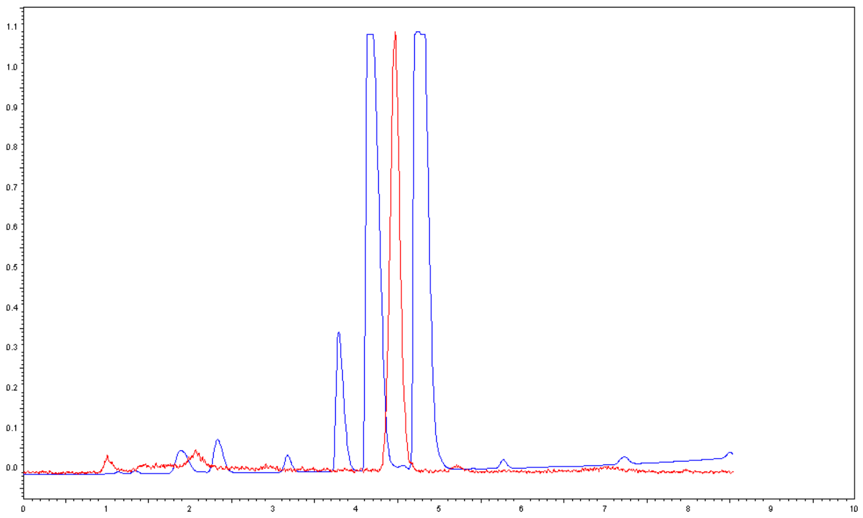

(b)

Figure S9. Radio-TLC (**a**) and radio-HPLC (**b**) of [^{18}F]FNOS-intermediate (**7**) radiolabeling reaction (HPLC condition: Altima C18 250 × 4.6 mm, linear gradient from 30% MeCN/70% water/0.1% TFA to 80% MeCN/20% water/0.1% TFA over 7 min, 2 mL/min, 254 nm). UV: Blue; Radioactivity: Red.

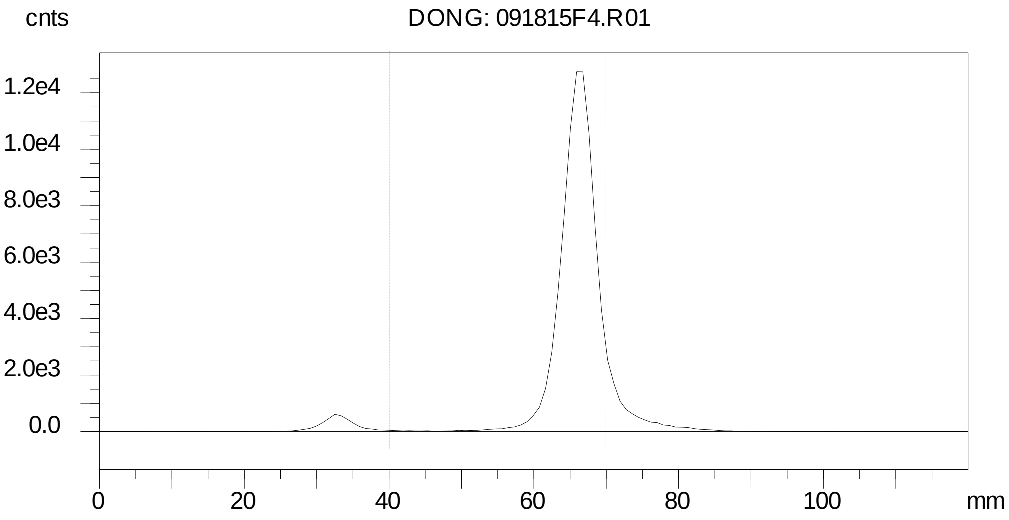

(**a**)

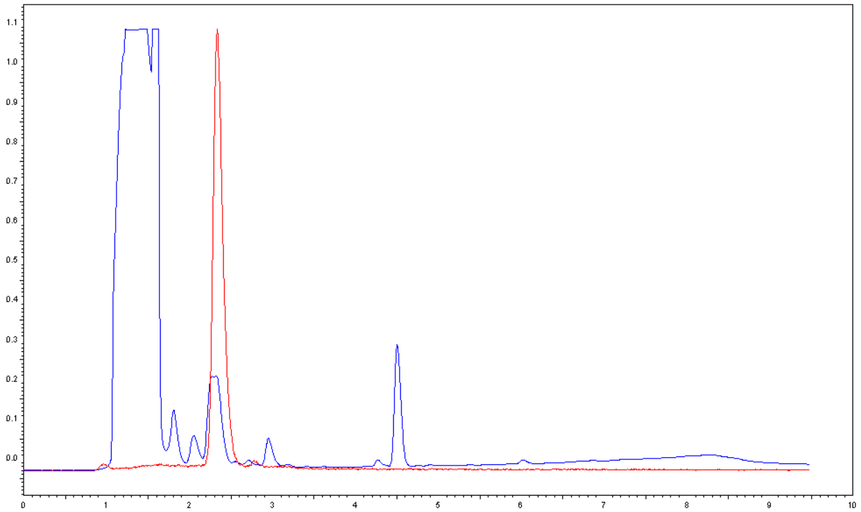

(b)

Figure S10. Radio-TLC (**a**) and radio-HPLC (**b**) of [^{18}F]FTT (**8**) radiolabeling reaction (HPLC condition: Altima C18 250 × 4.6 mm, linear gradient from 30% MeCN/70% water/0.1% TFA to 80% MeCN/20% water/0.1% TFA over 7 min, 2 mL/min, 254 nm). UV: Blue; Radioactivity: Red.

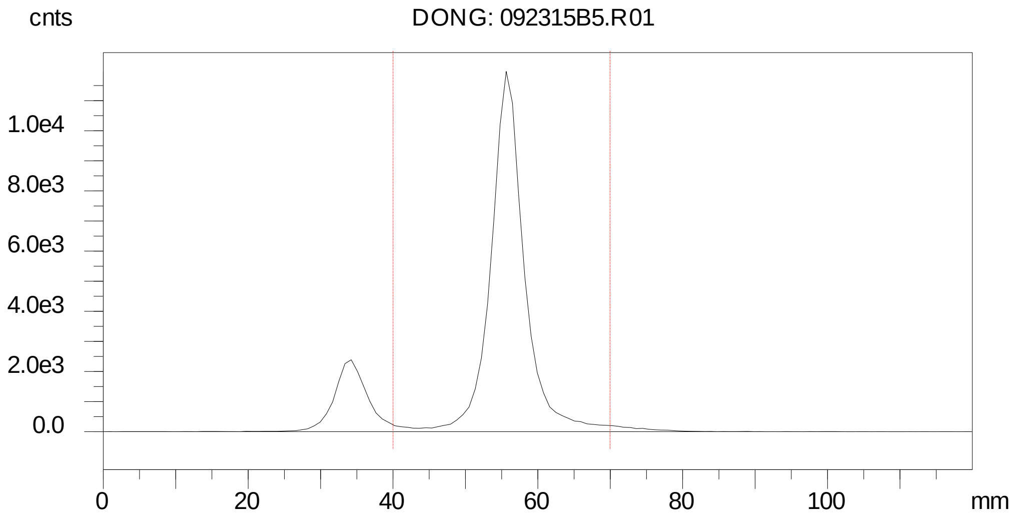

(**a**)

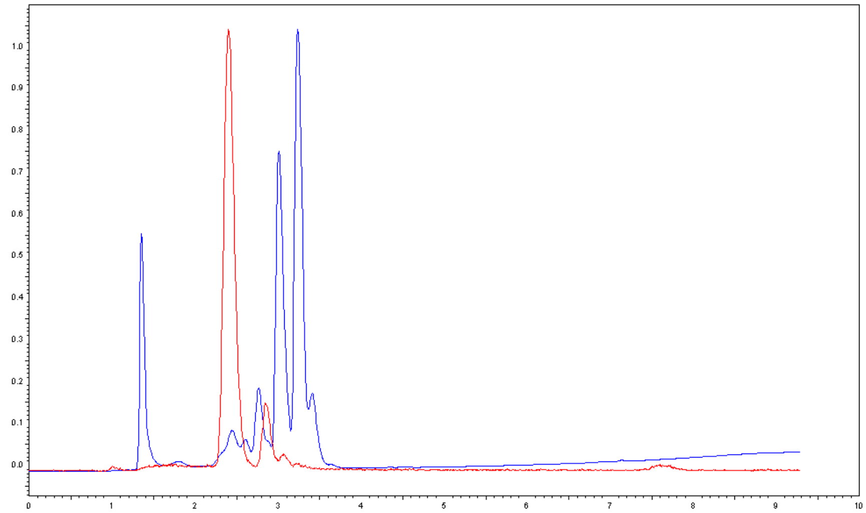

(b)

Figure S11. Radio-TLC (**a**) and radio-HPLC (**b**) of [^{18}F]FTT (**9**) radiolabeling reaction using bromine as leaving group. (HPLC condition: Altima C18 250 × 4.6 mm, linear gradient from 30% MeCN/70% water/0.1% TFA to 80% MeCN/20% water/0.1% TFA over 7 min, 2 mL/min, 254 nm). UV: Blue; Radioactivity: Red.

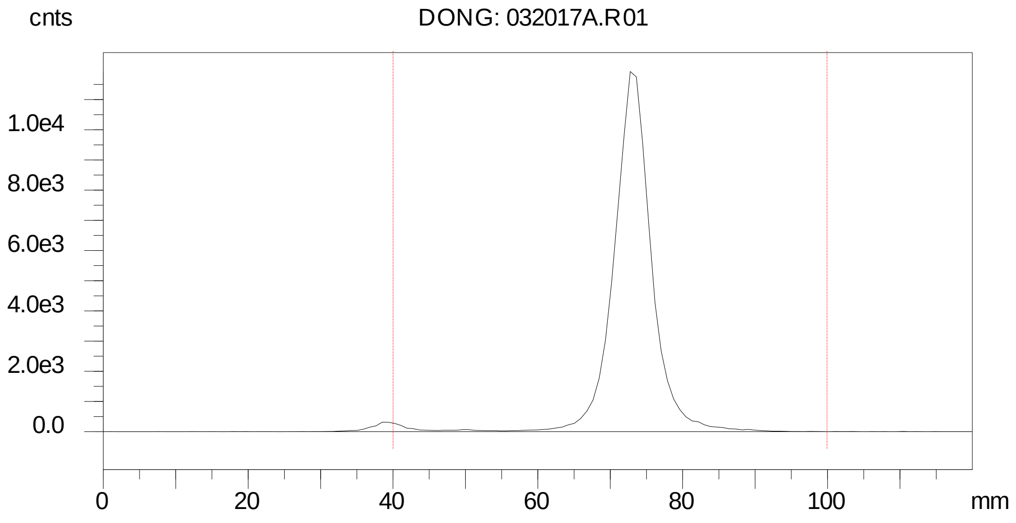

(**a**)

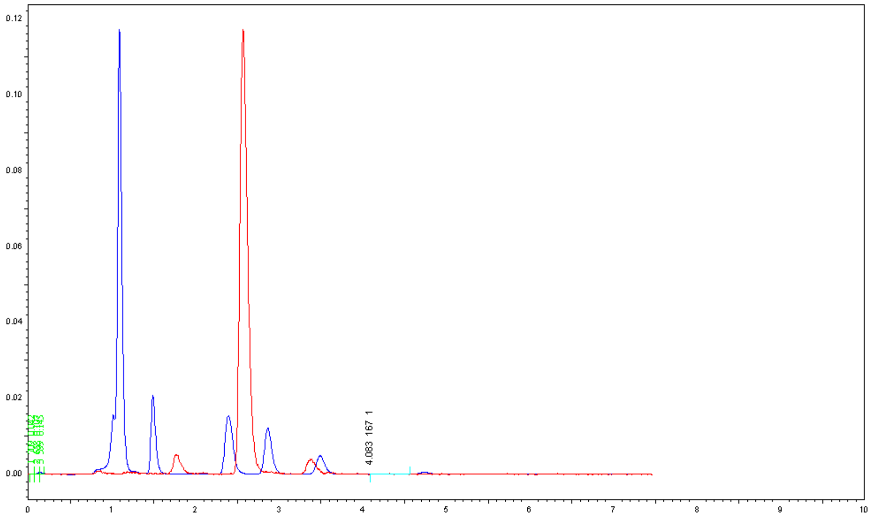

(b)

Figure S12. Radio-TLC (**a**) and radio-HPLC (**b**) of [^{18}F]FBAL (**9**) radiolabeling reaction (HPLC condition: Altima C18 250 × 4.6 mm, 60% MeCN/40% water/0.1% TFA, 2 mL/min, 254 nm). UV: Blue; Radioactivity: Red.

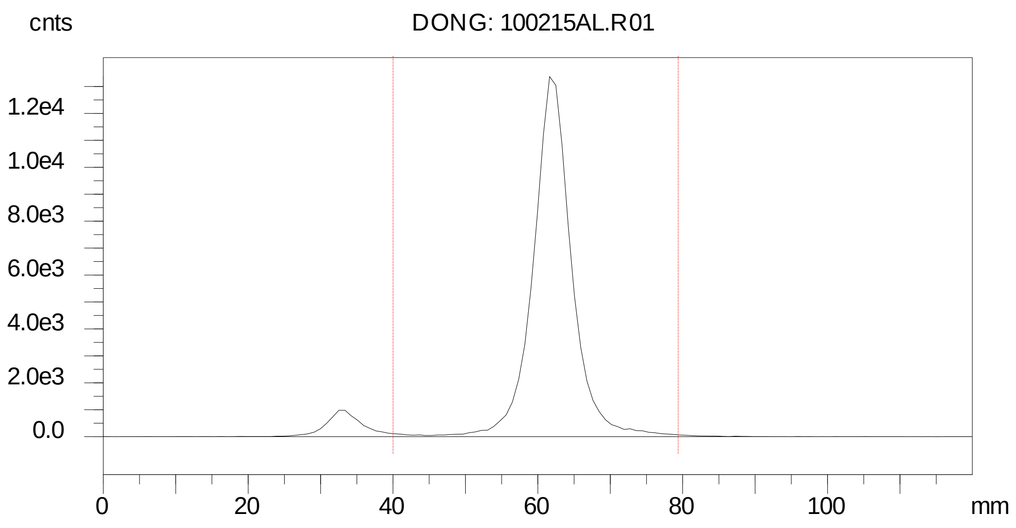

(**a**)

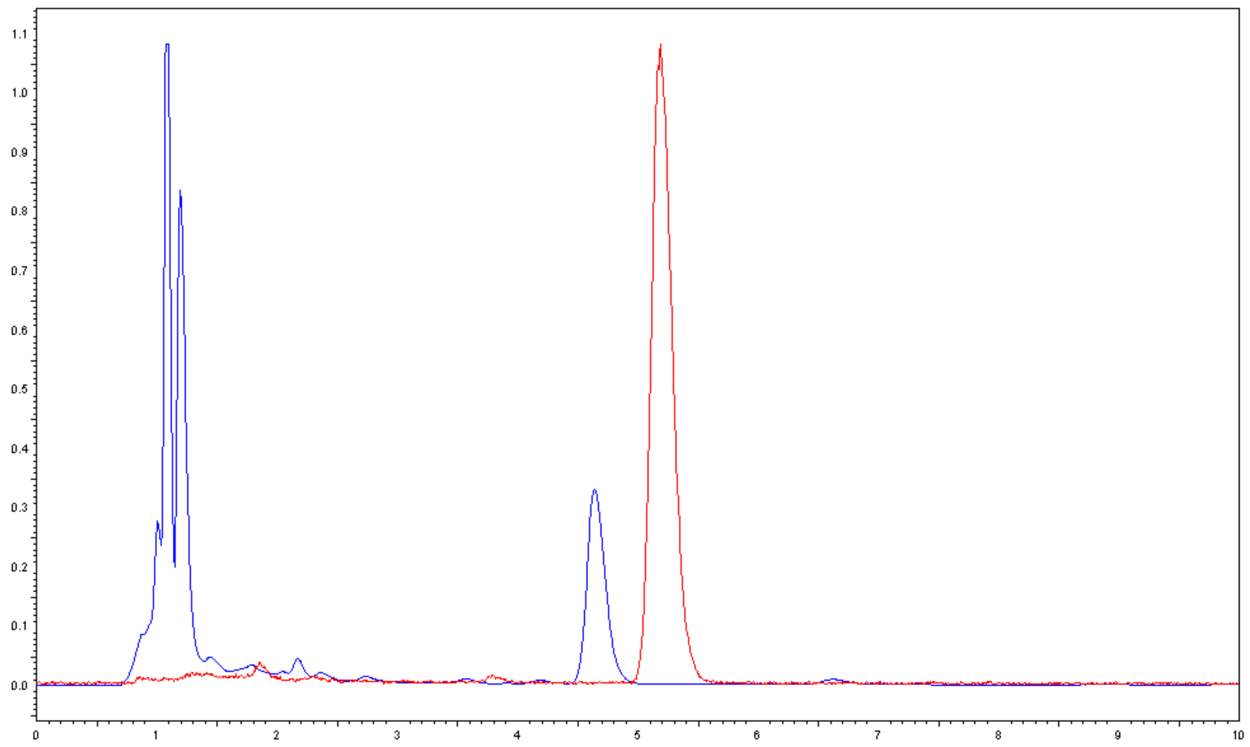

(b)

Figure S13. Radio-TLC (**a**) and radio-HPLC (**b**) of [^{18}F]FBA (**10**) radiolabeling reaction (HPLC condition: Altima C18 250 × 4.6 mm, 60% MeCN/40% water/0.1% TFA, 2 mL/min, 254 nm). UV: Blue; Radioactivity: Red.

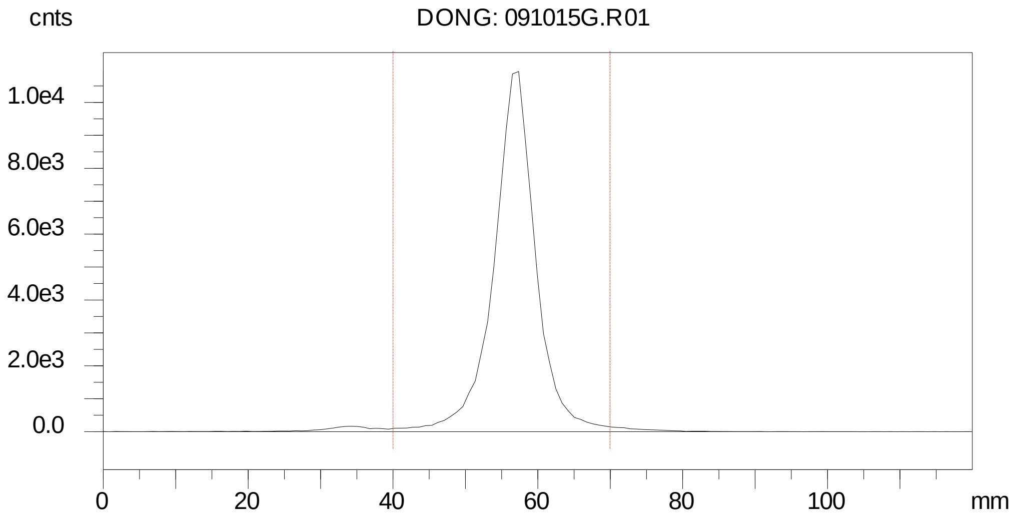

(**a**)

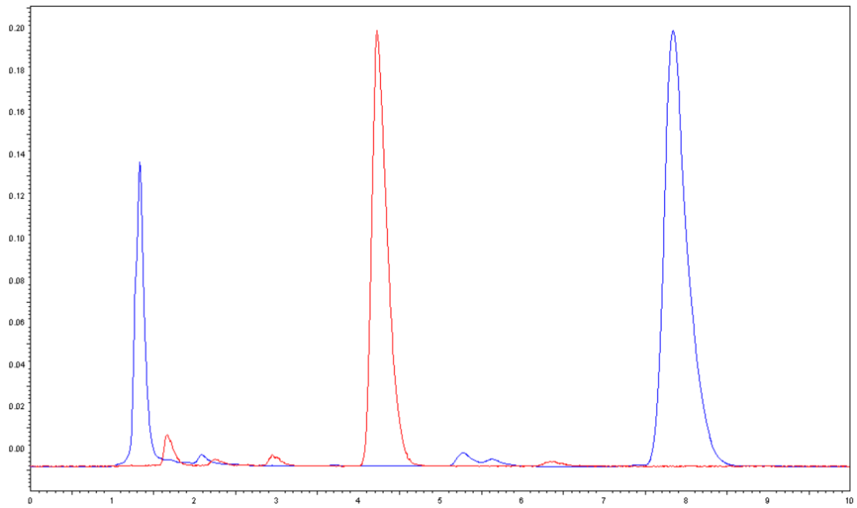

(b)

Figure S14. Radio-TLC (**a**) and radio-HPLC (**b**) of [^{18}F]FETs (**11**) radiolabeling reaction (HPLC condition: Altima C18 250 × 4.6 mm, 60% MeCN/40% water/0.1% TFA, 1 mL/min, 254 nm). UV: Blue; Radioactivity: Red.

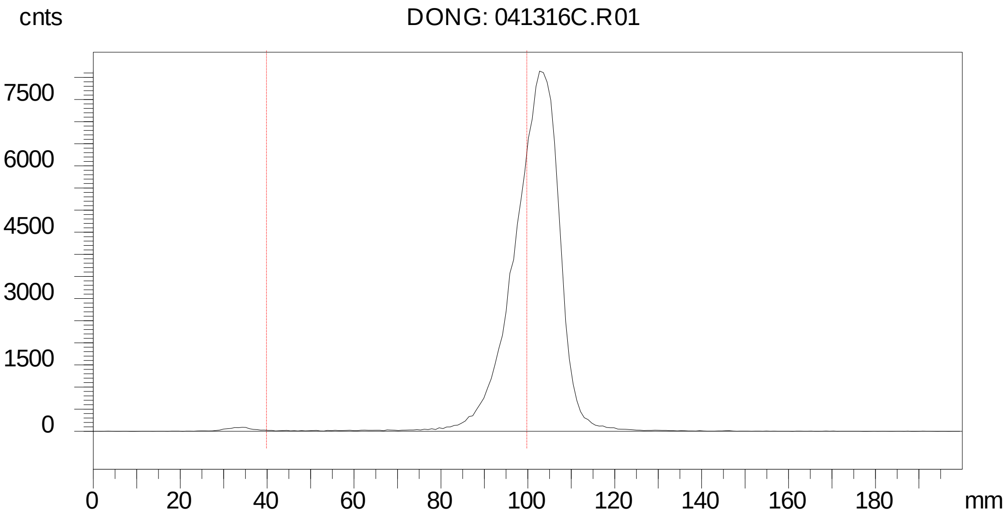

(**a**)

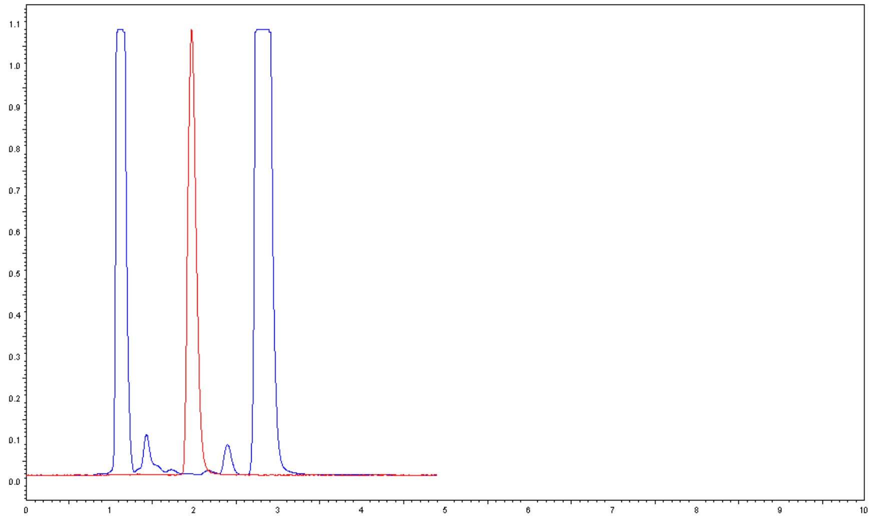

(b)

Figure S15. Radio-TLC (**a**) and radio-HPLC (**b**) of [^{18}F]FEAz (**12**) radiolabeling reaction (HPLC condition: Altima C18 250 × 4.6 mm, 60% MeCN/40% water/0.1% TFA, 2 mL/min, 254 nm). UV: Blue; Radioactivity: Red. Note: Radio-TLC was determined using a capillary tube filled with silica gel due to the violability of [^{18}F]FEAz.

Radiosynthesis of [^{18}F]FFNP (6)

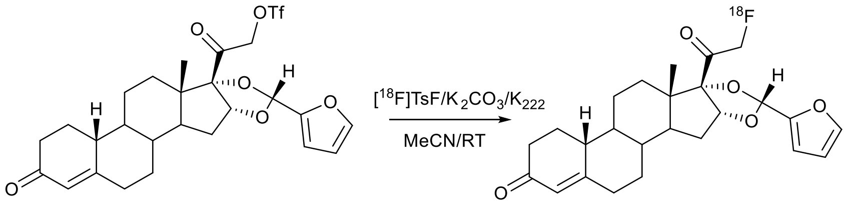

Into a 9 mL Pyrex tube containing K_2CO_3/K_{222} (2.5 mg, 2.8 μmol) was added [^{18}F]TsF (0.8 GBq) in acetonitrile (0.5 mL). The tube was capped, heated at 105 °C for 2.5 min and allowed to cool down for 3 min before adding the FFNP triflate precursor (2 mg, 3.6 μmol) in acetonitrile (100 μL). The tube was shaken briefly, and the reaction was allowed at room temperature for 6 min before the addition of a solution of ammonium formate (0.1 M, pH = 4.5) (1 mL) for HPLC purification (Column: Phenomenex Luna C18 250 × 10 mm, 5 μm; Mobile phase: 54% acetonitrile/46% water; Flow rate: 4 mL/min; UV: 250 nm). [^{18}F]FFNP (0.56 GBq, 89.5% RCY) was collected at 24-26 min and further processed using standard solid-phase extraction protocol to produce the final dose in 10% ethanol/saline for animal study.

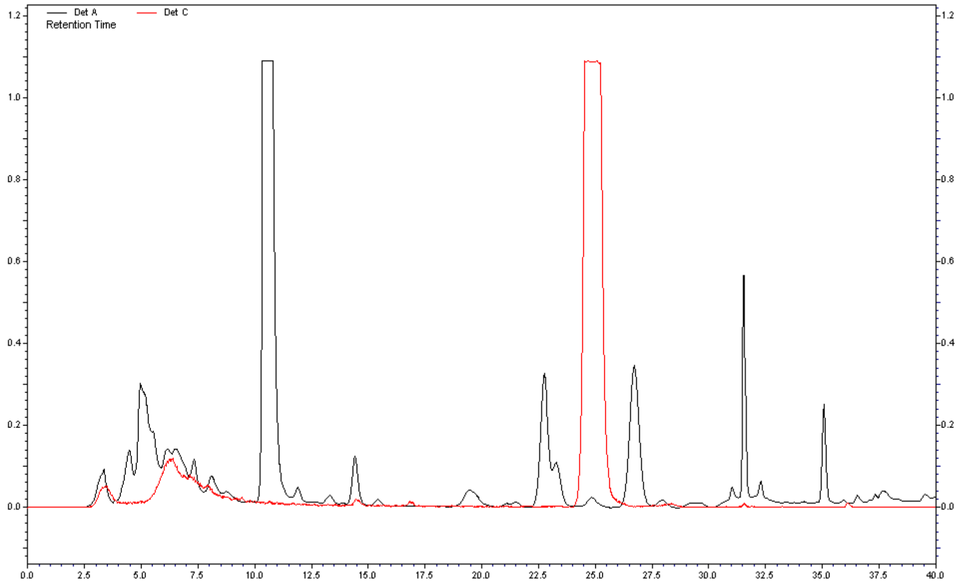

Figure S16. Semi-preparative radio-HPLC chromatography of [^{18}F]FFNP purification (Black: UV; Red: Radioactivity). [^{18}F]FFNP was collected at 24-26 min.

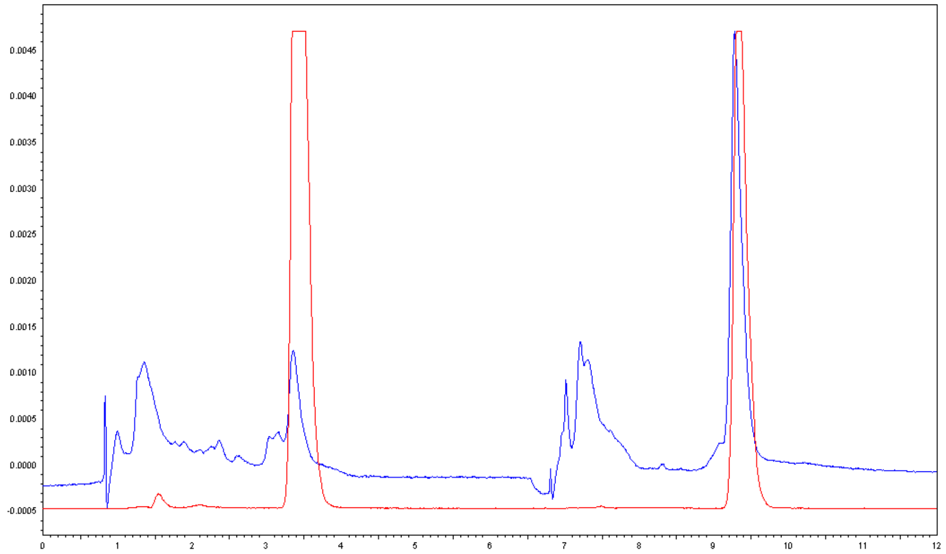

Figure S17. Analytical radio-HPLC chromatography of [^{18}F]FFNP (Blue: UV; Red: Radioactivity). The first injection is the animal dose and the second injection is the co-injection of authentic FFNP (HPLC condition: Altima C18 250 × 4.6 mm, 70% MeCN/30% water, 250 nm, 2 mL/min).

Radiosynthesis of [^{18}F]FTT (8)

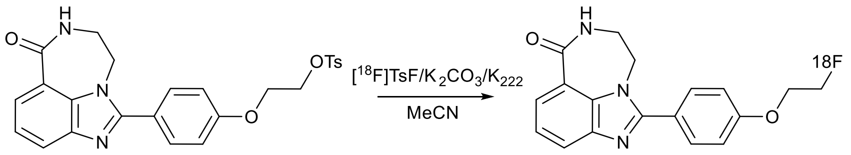

Into a 9 mL Pyrex tube containing the FTT tosylate precursor (1 mg, 2.1 µmol) and K_2CO_3/K_{222} (2.5 mg, 2.8 µmol) as solids was added a solution of [^{18}F]TsF(1.1 GBq) in acetonitrile (0.5 mL). The tube was capped and then heated at 105 °C for 12 min before the addition of 0.1% TFA in water (4 mL) for HPLC purification (Column: Agilent SB-C18 250 × 9.4 mm, 5 µm; Mobile phase: 17% acetonitrile/83% water with 0.1% TFA; Flow rate: 4 mL/min; UV: 250 nm). [^{18}F]FTT (0.68 GBq, 79% RCY) was collected at 17-18 min, and further processed using the typical solid-phase extraction method to produce the final dose for animal study in 10 % ethanol/saline.

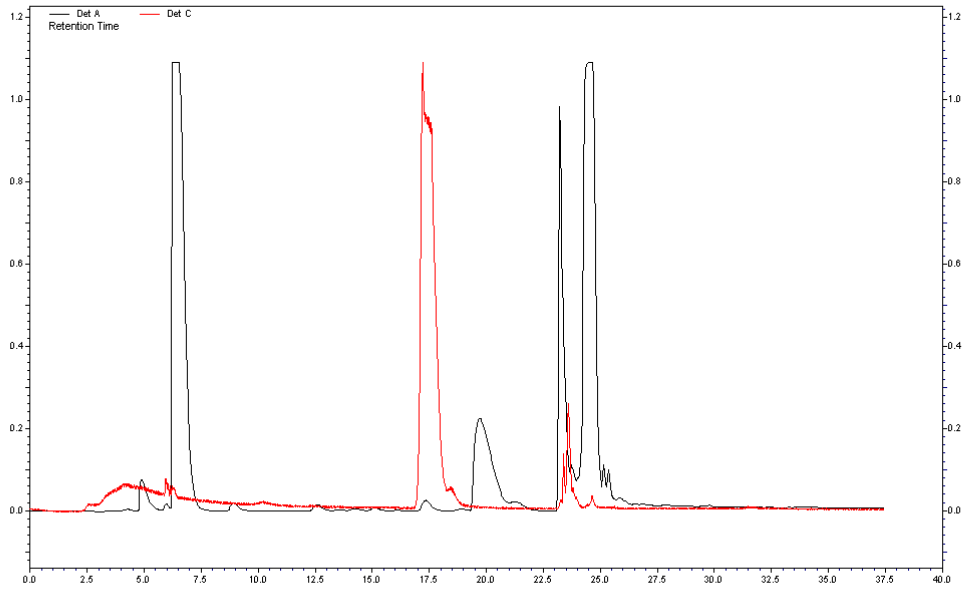

Figure S18. Semi-preparative radio-HPLC chromatography of [18F]FTT purification (Black: UV; Red: Radioactivity). Note: After the collection of [18F]FTT, the column was rinsed with 80% MeCN/20% water/0.1% TFA. [18F]FTT was collected at 17-18 min.

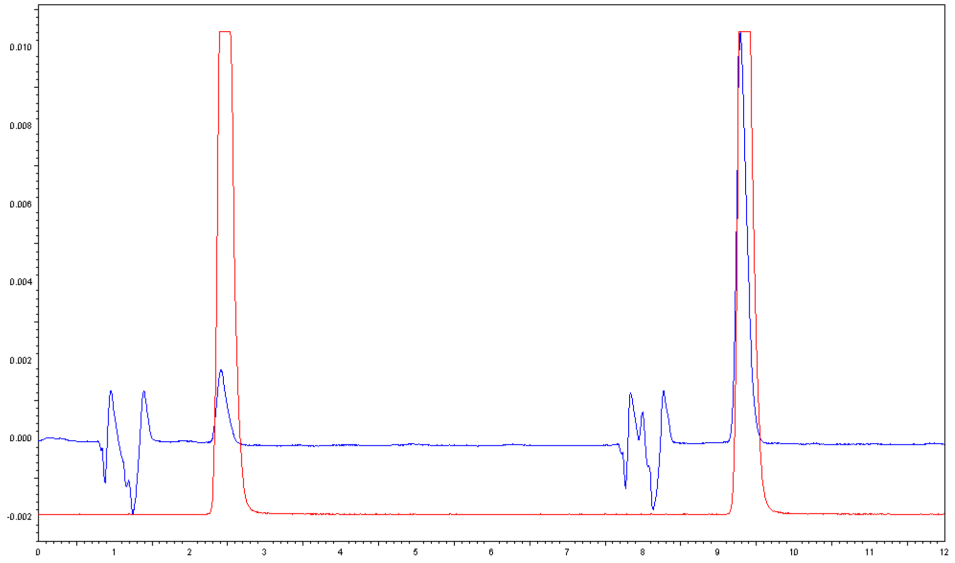

Figure S19. Analytical radio-HPLC chromatography of [18F]FTT (Blue: UV; Red: Radioactivity). The first injection is the animal dose and the second injection is the co-injection of FTT. (HPLC condition: Altima C18 250 × 4.6 mm, 30% MeCN/70% water, 250 nm, 2 mL/min)

One-pot radiosynthesis of [^{18}F]FDHT (13)

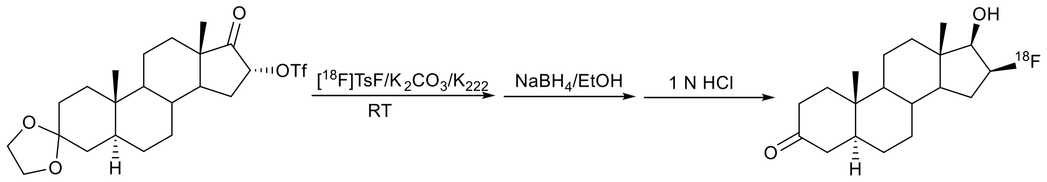

Into a 9 mL Pyrex tube containing K_2CO_3/K_{222} (1.8 mg, 2.0 µmol) was added a solution of [^{18}F]TsF(1.6 GBq) in acetonitrile (0.5 mL). After the tube was capped and heated at 110 °C for 3 min, the tube was removed from the oil bath and allowed to cool down in 2 min. Into the tube was then added the FDHT precursor (0.9 mg) in acetonitrile (100 µL), and the reaction was allowed at room temperature for 7 min. After a solution of $NaBH_4$ (5 mg) in ethanol (500 µL) was added, the reaction mixture was heated at 80 °C for 3 min, followed by the addition of 1N HCl (500 µL) and heated at 80 °C for 5 min. The reaction mixture was diluted with water (2 mL) and 1N NaOH (500 µL) for HPLC purification (Column: Phenomenex Luna C18 250 × 10 mm, 5 µm; Mobile phase: 45% acetonitrile/55% water; Flow rate: 4 mL/min; UV: 214 nm). [^{18}F]FDHT (0.78 GBq, 74% RCY) was collected at 21-22 min, and further processed using the typical solid-phase extraction method to produce the final dose for animal study in 10% ethanol/saline.

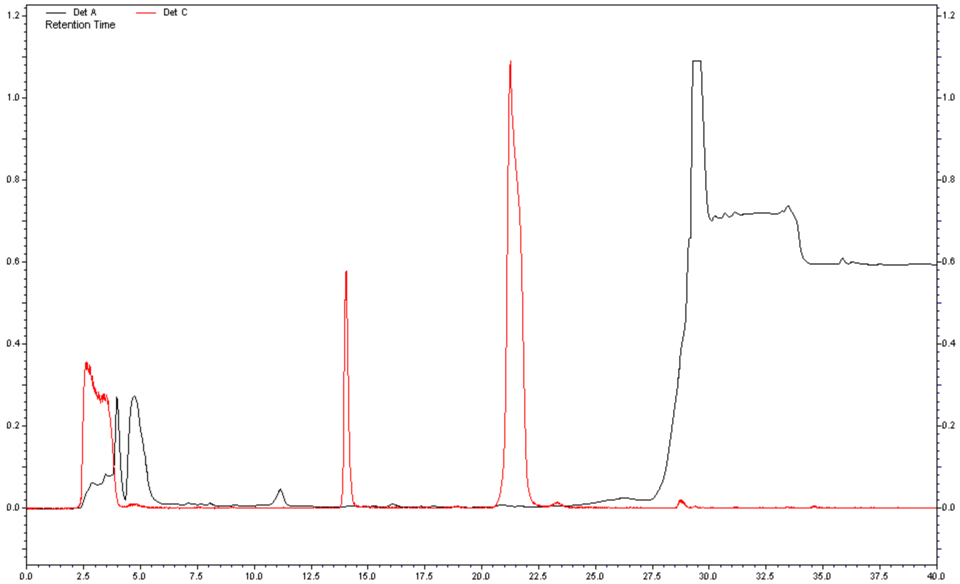

Figure S20. Semi-preparative HPLC chromatography of [^{18}F]FDHT purification (Black: UV; Red: Radioactivity). [^{18}F]FDHT was collected at 21-22 min. Note: After the collection of [18F]FDHT, the column was rinsed with 80% MeCN/20% water.

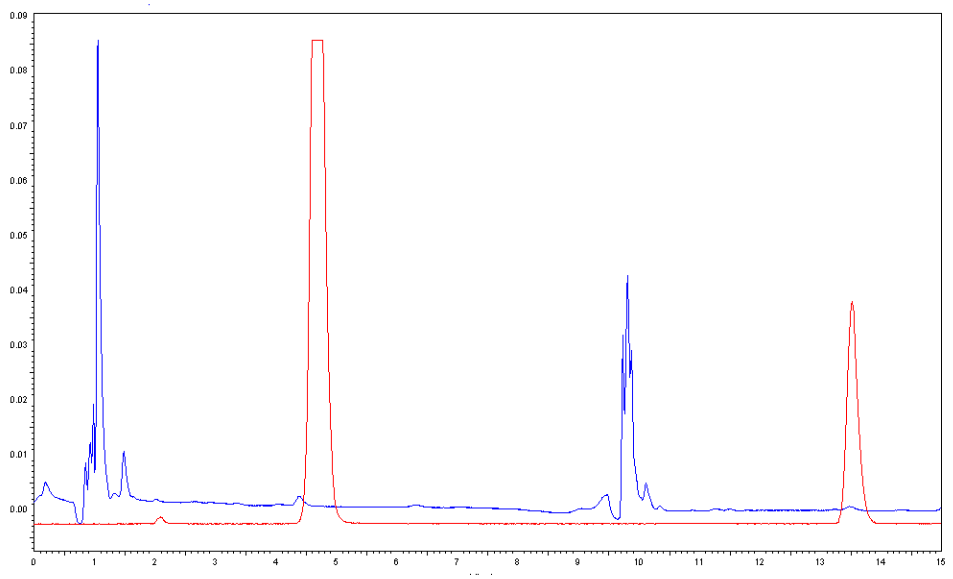

Figure S21. Analytical HPLC chromatography of [^{18}F]FDHT (Blue: UV; Red: Radioactivity). The first injection is the animal dose and the second injection is the co-injection of the authentic FDHT (HPLC condition: Altima C18 250 × 4.6 mm, 55% MeCN/45% water, 215 nm, 2 mL/min).

Radiosynthesis of [^{18}F]BCPP-EF (14)

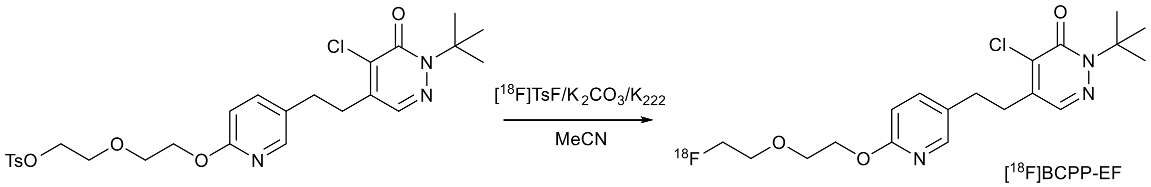

Into a 9 mL Pyrex tube containing K_2CO_3/K_{222} (3.4 mg, 3.8 µmol) was added [^{18}F]TsF (1.1 GBq) in acetonitrile (0.5 mL). The tube was capped and heated at 105 °C for 2 min before the addition of the precursor (2.6 mg) in acetonitrile (100 µL). The reaction mixture was heated at 95 °C for 12 min and then diluted with water with 0.1% TFA (1 mL) for HPLC purification (Column: Phenomenex Luna C18 250 × 10 mm, 5 µm; Mobile phase: 50% acetonitrile/50% water with 0.1% TFA; Flow rate: 4 mL/min; UV: 275 nm). [^{18}F]BCPP-EF (0.41GBq, 44.9% RCY) was collected at 17-18 min and further processed using the standard solid-phase extraction protocol to produce the final dose in 10% ethanol/saline for animal study.

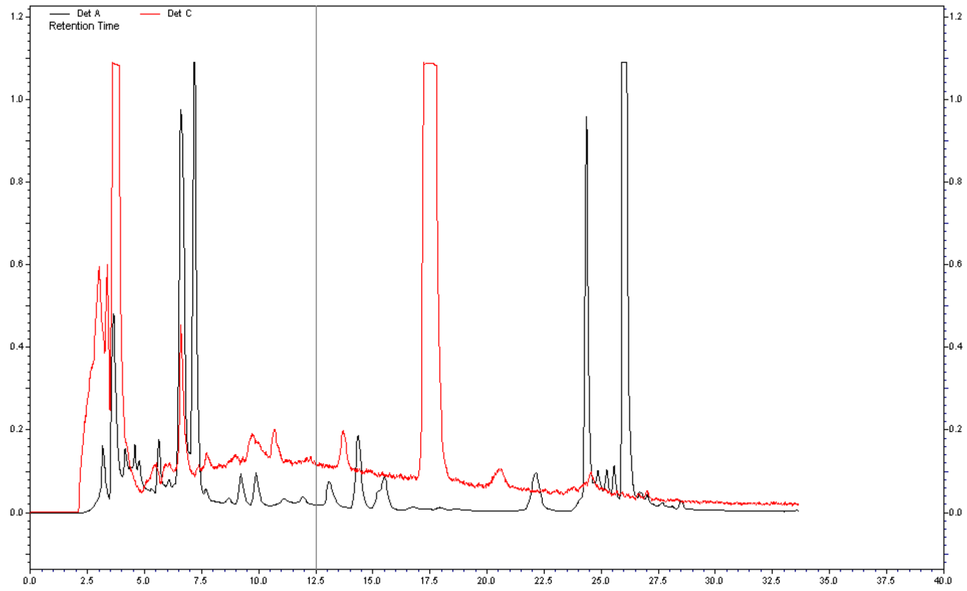

Figure S22. Semi-preparative radio-HPLC chromatography of [^{18}F]BCPP-EF purification (Black: UV; Red: Radioactivity). After the collection of [^{18}F]BCPP-EF, the column was rinsed with 80% MeCN/20% water/0.1% TFA. [^{18}F]BCPP-EF was collected at 17-18 min.

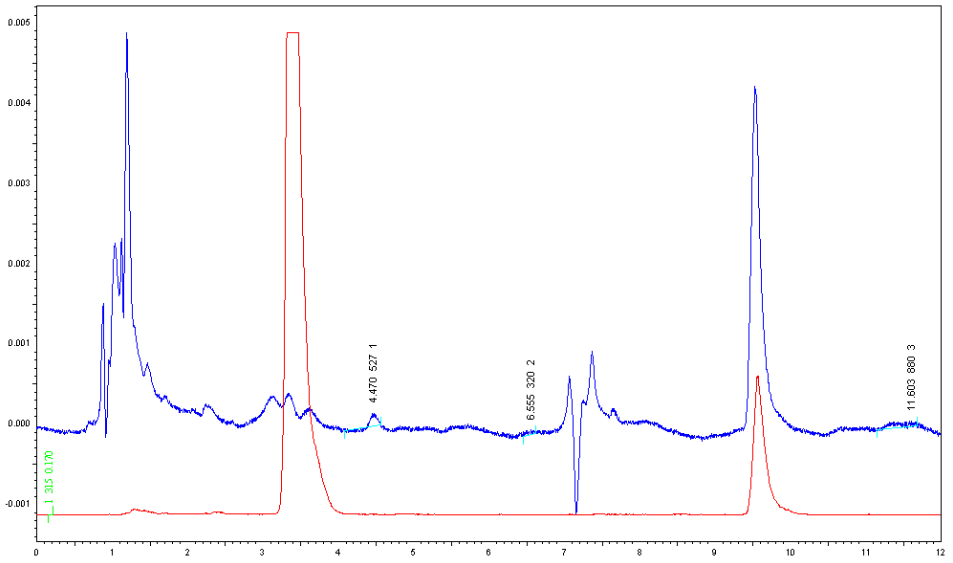

Figure S23. Analytical radio-HPLC chromatography of [^{18}F]BCPP-EF (Blue: UV; Red: Radioactivity). The first injection is the animal dose and the second injection is the co-injection of authentic BCPP-EF (HPLC condition: Altima C18 250 × 4.6 mm, 60% MeCN/40% water/0.1% TFA, 275 nm, 2 mL/min).

Radiosynthesis of [^{18}F]FMISO (15)

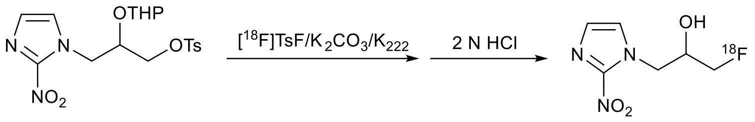

Into a 9 mL Pyrex tube containing K_2CO_3/K_{222} (4.3 mg, 4.8 µmol) was added a solution of [^{18}F]TsF(0.61 GBq) in acetonitrile (1.5 mL). After the tube was capped and heated at 102 °C for 2 min, a solution of the precursor NITTP (2 mg) in acetonitrile (100 µL) was added. The solution was heated at 102 °C for 10 min and then concentrated under an argon flow to 0.5 mL before the addition of 2 N HCl (500 µL) for deprotection at 102 °C for 7 min. Upon completion, the reaction mixture was diluted with a solution of ammonium formate (AMF) (0.1 M) with 5 N NaOH (200 µL) for HPLC purification (Column: Phenomenex Luna C18 250 × 10 mm, 5 µm; Mobile phase: 10% acetonitrile/90% 0.1 M AMF buffer (pH = 6.5); Flow rate: 4 mL/min; UV: 254 nm). [^{18}F]FMISO (0.30 GBq, 71 RCY) was collected at 12-14 min, and further processed using the typical solid-phase extraction method (using a Waters HLB plus cartridge) to produce the final dose for animal study in 10% ethanol/saline.

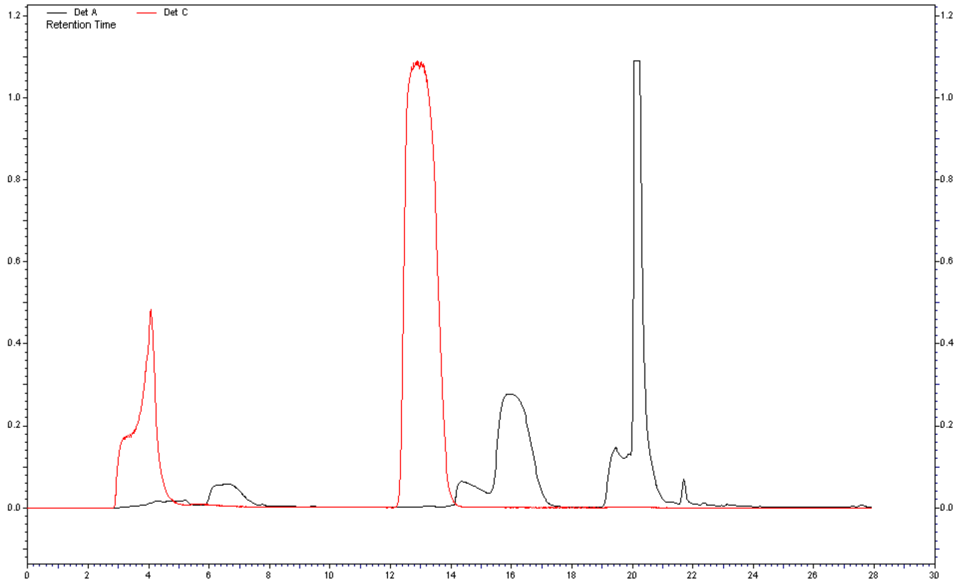

Figure S24. Semi-preparative radio-HPLC chromatography of [^{18}F]FMISO purification (Black: UV; Red: Radioactivity). After the collection of [^{18}F]BCPP-EF, the column was rinsed with 80% MeCN/20% water/0.1% TFA. [^{18}F]FMISO was collected at 12-14 min.

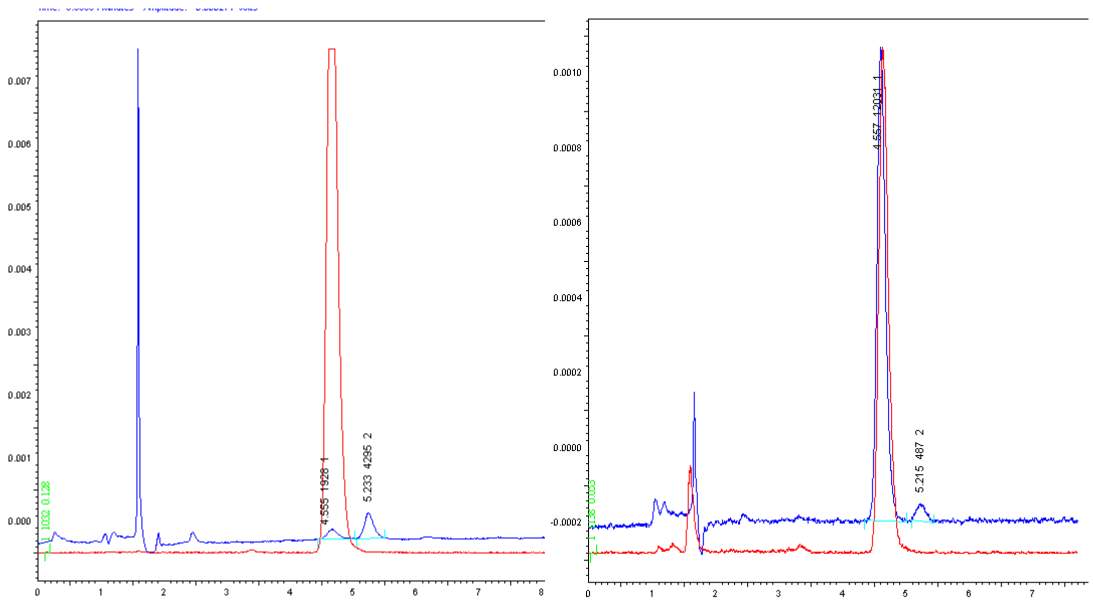

Figure S25. Analytical radio-HPLC chromatography of [^{18}F]FMISO (Blue: UV; Red: Radioactivity). The first injection is the animal dose and the second injection is the co-injection of FMISO (HPLC condition: Altima C18 250 × 4.6 mm, 10% MeCN/90% AMF 0.1 M pH = 6.5, 254 nm, 2 mL/min).

Radiosynthesis of [^{18}F]FTHA (16)

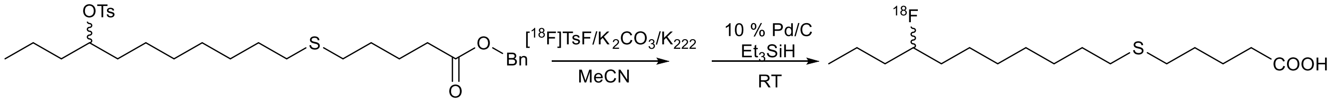

Into a 9 mL Pyrex tube containing K_2CO_3/K_{222} (2.5 mg, 2.8 μmol) was added [^{18}F]TsF (1.41 GBq) in acetonitrile (1 mL). The tube was capped, heated at 105 °C for 2.5 min and then acetonitrile was removed under an argon flow at 105 °C, followed by the addition of the precursor (2 mg) in acetonitrile (0.5 mL). After the reaction was heated at 105 °C for 8 min, the solution passed through a silica gel plug (10 × 5 mm) to remove unreacted [^{18}F]fluoride, followed by rinsing the tube and the plug with acetonitrile (2 × 0.5 mL). For deprotection, 10% Pd/C (6 mg) and Et_3SiH (10 μL) in methanol (0.5 mL) were added to the combined solution. After 7 min at room temperature, the reaction mixture passed through a 3 mL cartridge containing celite (10 mm) to remove Pd/C, followed by rinsing with methanol (2 × 0.5 mL). All the eluted solution was combined and diluted with water (1 mL) for HPLC purification (Column: Phenomenex Luna C18 250 × 10 mm, 5 μm; Mobile phase: 85% methanol/15% water with 0.4% acetic acid; Flow rate: 4 mL/min; UV: 250 nm). [^{18}F]FTHA (0.41 GBq, 44.9% RCY) was collected at 12-14 min and further processed using standard solid-phase extraction protocol (PBS buffer containing ascorbic acid 1 mg/10 mL was used) to produce the final dose in 10% ethanol/saline for animal study.

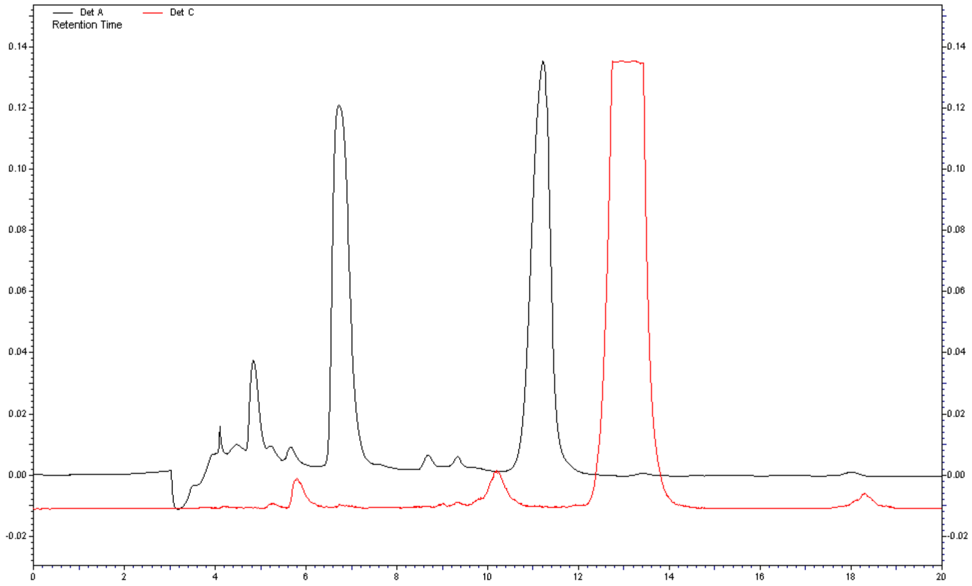

Figure S26. Semi-preparative radio-HPLC chromatography of [^{18}F]FTHA purification (Black: UV; Red: Radioactivity). [^{18}F]FTHA was collected at 12-14 min.

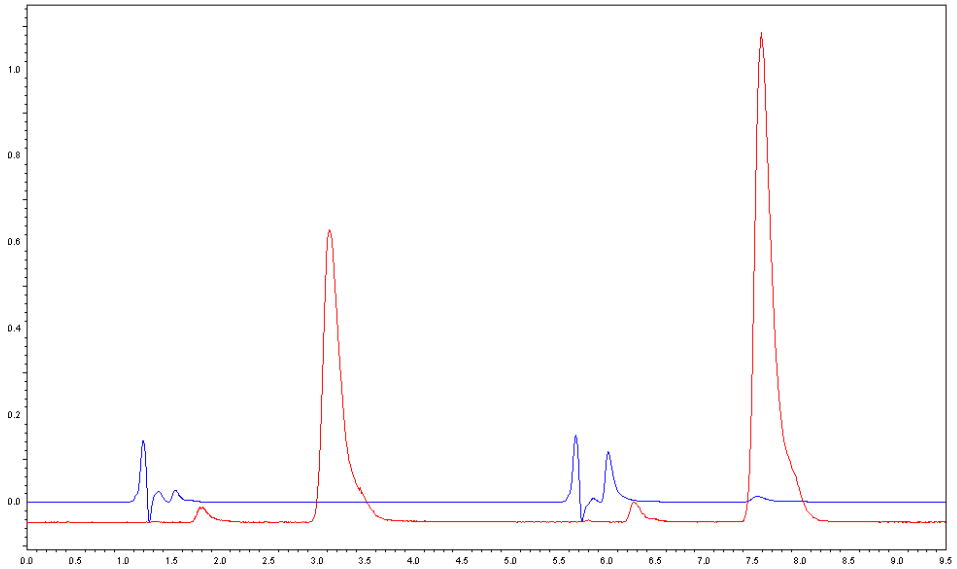

Figure S27. Analytical radio-HPLC chromatography of [^{18}F]FTHA (Blue: UV; Red: Radioactivity). The first injection is the animal dose and the second injection is the co-injection of FTHA (HPLC condition: Altima C18 250 × 4.6 mm, 90% methanol/10% water with 0.4% acetic acid, 230 nm, 2 mL/min).

Radiosynthesis of [^{18}F]FGLN (17)

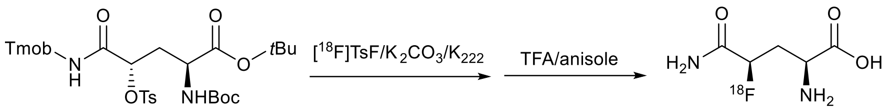

The synthesis followed the reported procedure with some modification in the deprotection step. Into a 9 mL Pyrex tube containing K_2CO_3/K_{222} (1.6 mg, 1.8 μmol) was added a solution of [^{18}F]TsF(1.7 GBq) in acetonitrile (1 mL). After the tube was capped and heated at 110 °C for 3 min, the solution was concentrated to < 50 μL under an argon flow at 110 °C before the addition of the FGLN precursor (2 mg) in acetonitrile (0.3 mL). The reaction mixture was heated at 70 °C for 8 min, and then passed through a Waters silica light cartridge to remove unreacted [^{18}F]fluoride, followed by rinsing the tube and the cartridge with acetonitrile (1.5 mL). The eluted solution was combined and diluted with water (3 mL) for HPLC purification (Column: Agilent SB-C18 250 × 9.4 mm, 5 μm; Mobile phase: 60% acetonitrile/40% water with 0.1% TFA; Flow rate: 4 mL/min; UV: 261 nm). The ^{18}F-labeled intermediate of FGLN (0.69 GBq, 54% RCY) was collected at 15-16 min, and then the collected fraction was diluted with water (30 mL) and passed through a Waters HLB light cartridge, followed by rinsing the cartridge with water (5 mL). The radioactivity was eluted from the cartridge and dried by passing through 3 mL cartridge (containing from top to bottom: celite (10 mm), sodium sulfate (5 mm), magnesium sulfate (5 mm) and silica gel (2 mm)), using ethyl acetate (4 × 0.5 mL). After ethyl acetate was removed under an argon flow at 110 °C, TFA (500 μL) with anisole (5 μL) was added to the above residue. The solution was heated at 60 °C for 6 min, and then TFA was removed under an argon flow at room temperature to afford [^{18}F]FGLN (0.57 GBq) as residue, which was dissolved in ethanol (100 μL) and PBS buffer (1900 uL) for animal study.

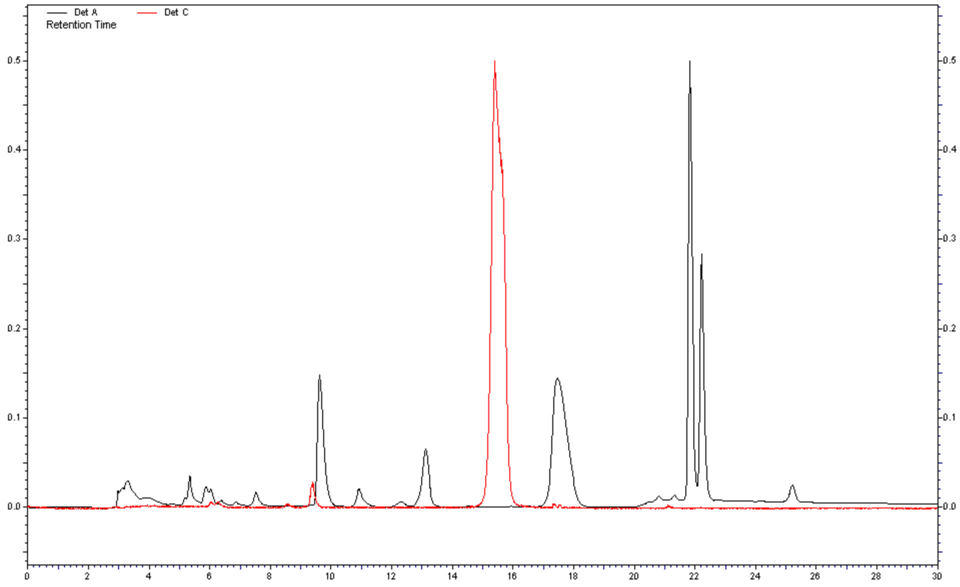

Figure S28. Semi-preparative radio-HPLC chromatography of [^{18}F]FGLN purification (Black: UV; Red: Radioactivity). [^{18}F]FGLN intermediate was collected 15-16 min.

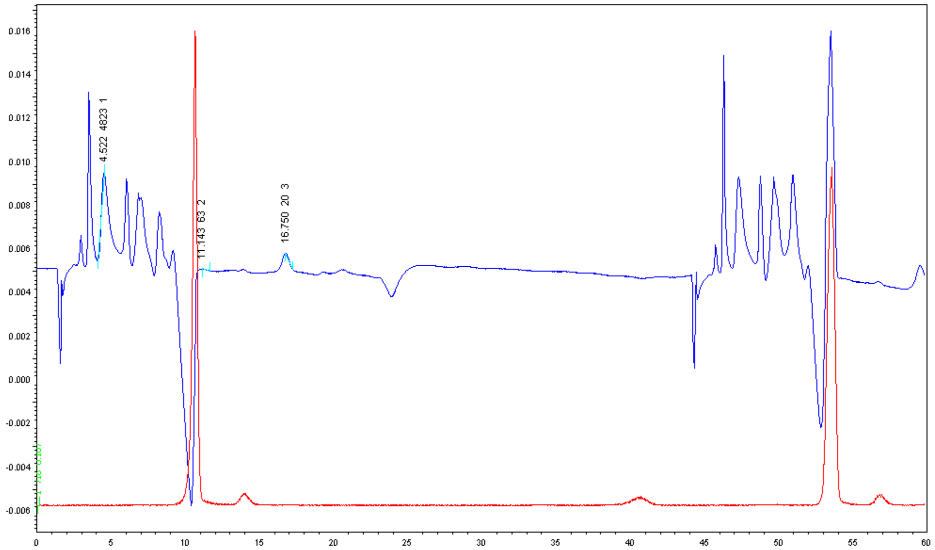

Figure S29. Analytical radio-HPLC chromatography of [^{18}F]FGLN (Blue: UV; Red: Radioactivity). The first injection is the animal dose and the second injection is the co-injection of FGLN(HPLC condition: Phenomenex Chirex 3126 (D)-penicillamine 250 × 4.6 mm, 1 mM CuSO4, 254 nm, 1 mL/min).

Synthesis of [^{18}F]FSPG (18)

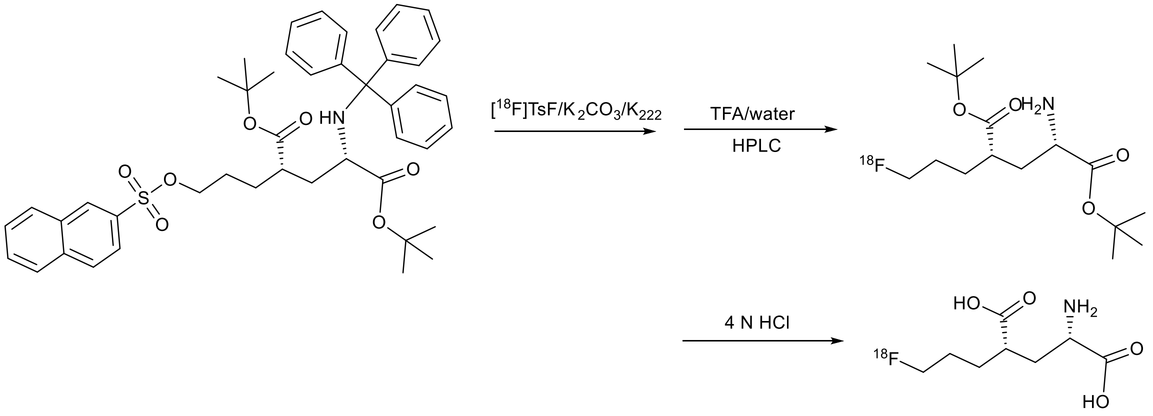

Into a 9 mL Pyrex tube containing K_2CO_3/K_{222} (1.7 mg, 1.9 µmol) was added [^{18}F]TsF (1.1 GBq) in acetonitrile (0.6 mL). The tube was capped and heated at 105 °C for 3 min before the addition of

the precursor (2 mg) in acetonitrile (100 µL). The reaction mixture was heated at 105 °C for 8 min and then treated with TFA (25 µL) in acetonitrile (300 µL) and water (100 µL), followed by dilution with water (3 mL) for HPLC purification (Column: Phenomenex Luna C18 250 × 10 mm, 5 µm; Mobile phase: 35% acetonitrile/65% water with 0.1% TFA; Flow rate: 4 mL/min; UV: 250 nm). The partially de-protected intermediate of [^{18}F]BCPP-EF (0.58 GBq, 60.4% RCY) was collected at 21-22 min and diluted with water (40 mL). The solution passed through a Waters C18 light cartridge under pressure and the cartridge was rinsed with water (10 mL) to remove residual solvent. The radioactivity was eluted with ethanol (0.5 ml) and ethanol was then removed under an argon flow at 105 °C before the addition of 4 N HCl (200 µL) for de-protection. The mixture was heated at 105 °C for 7 min and then diluted with water (1 mL) and neutralized with 1 N NaOH to pH = 5-7. The final dose was prepared by diluting with water to a total volume of 5.2 mL to achieve the concentration of sodium chloride (0.9%) for injection.

The analysis of the tracer was carried out using OPA reagent (Sigma Aldrich P0532) as visualization reagent as reported (Edwards, R., et al., Robust and Facile Automated Radiosynthesis of [^{18}F]FSPG on the GE FASTlab. *Mol Imaging Biol*, 2021. 23(6): p. 854-864.).

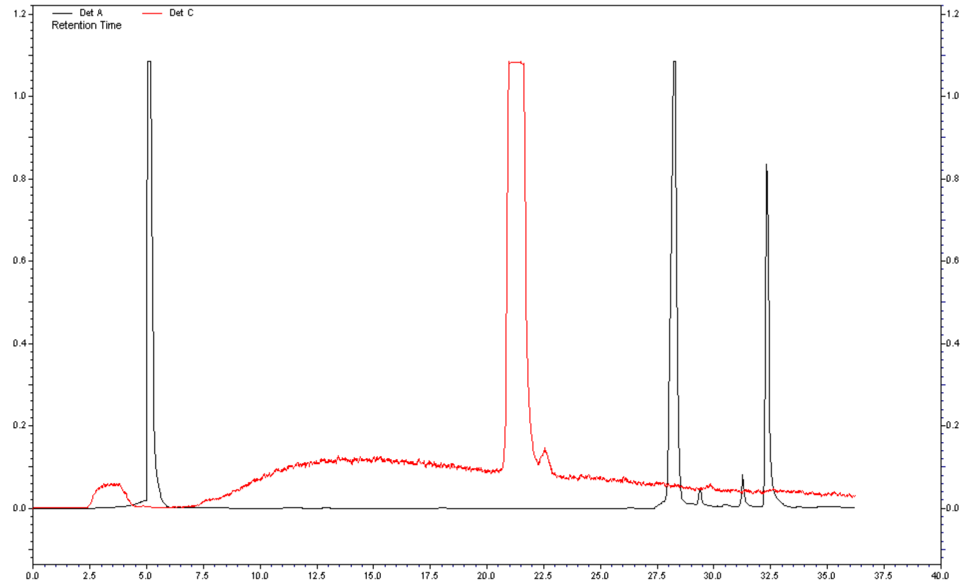

Figure S30. Semi-preparative radio-HPLC chromatography of [^{18}F]FSPG purification (Black: UV; Red: Radioactivity). [^{18}F]FSPG was collected at 20.5-22 min.

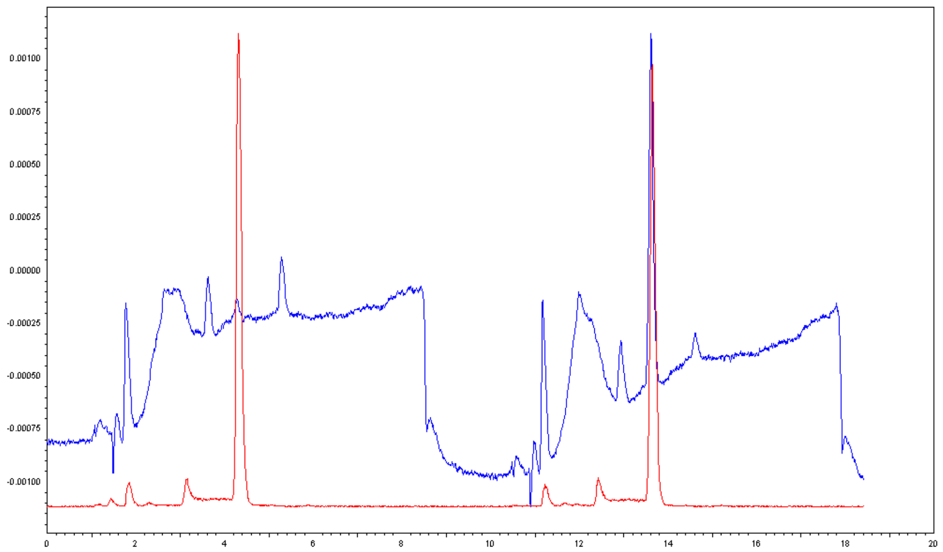

Figure S31. Analytical radio-HPLC chromatography of [^{18}F]FSPG, visualized with OPA reagent (Blue: UV; Red: Radioactivity). The first injection is the animal dose and the second injection is the co-injection of FSPG (HPLC condition: Altima C18 250 × 4.6 mm, linear gradient from 30% MeCN/70% water/0.1% TFA to 80% MeCN/20% water/0.1% TFA, 314 nm, 2 mL/min).

Condition: [^{18}F]FSPG (500 µL) + Phthaldialdehyde Reagent (25 µL), RT, up to 10 min.

Radiosynthesis of [^{18}F]DPA-714 (19)

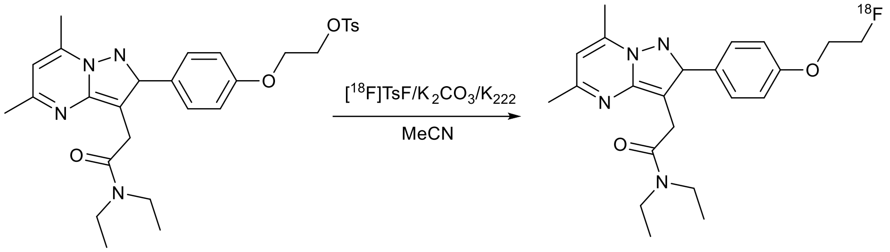

Into a 9 mL Pyrex tube containing K_2CO_3/K_{222} (1.8 mg, 2.0 µmol) and the precursor (1 mg, 1.8 µmol) was added [^{18}F]TsF (1.1 GBq) in acetonitrile (0.5 mL). The tube was capped and heated at 110 °C for 8 min before the addition of a solution of 0.1 M ammonium formate (pH = 4.5) (1 mL) for HPLC purification (Column: Phenomenex Luna C18 250 × 10 mm, 5 µm; Mobile phase: 61% methanol/39% 0.1 M ammonium formate buffer (pH = 4.5); Flow rate: 4 mL/min; UV: 250 nm). [^{18}F]DPA-714 (0.69 GBq, 74.4% RCY) was collected at 23-25 min and further processed using standard solid-phase extraction protocol to produce the final dose in 10% ethanol/saline for animal study.

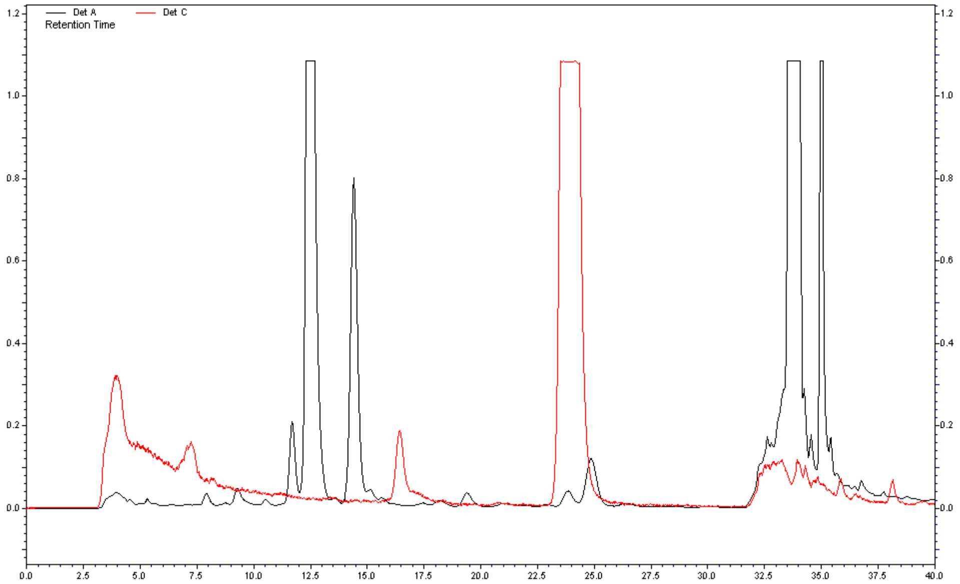

Figure S32. Semi-preparative radio-HPLC chromatography of [^{18}F]DPA-714 purification (Black: UV; Red: Radioactivity). [^{18}F]DPA-714 was collected at 23-25 min.

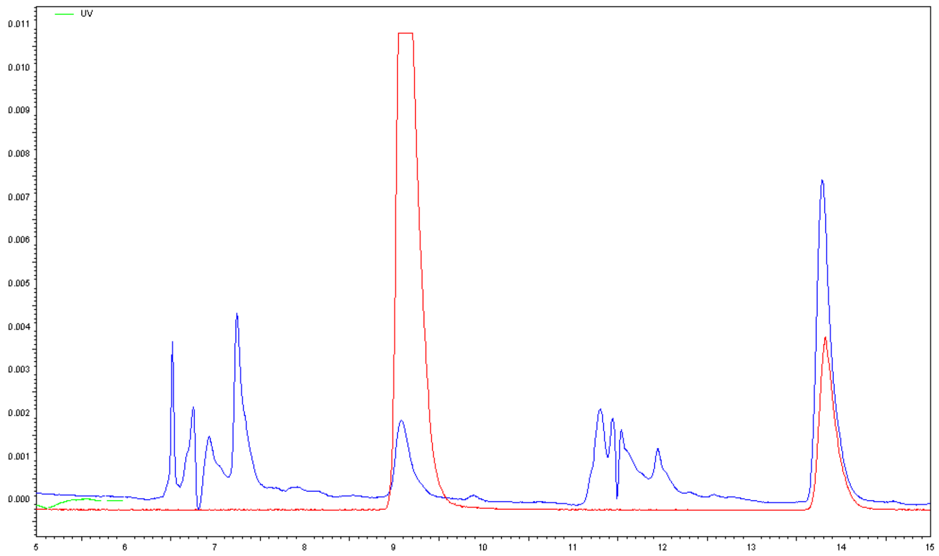

Figure S33. Analytical radio-HPLC chromatography of [^{18}F]DPA-714 (Blue: UV; Red: Radioactivity). The first injection is the animal dose and the second injection is the co-injection of DPA-714 (HPLC condition: Altima C18 250 × 4.6 mm, 60% MeCN/40% water/0.1% TFA, 254 nm, 2 mL/min).

.

Radiosynthesis of 4-[^{18}F]fluorobenzaldehyde (9) for the radiosynthesis of [^{18}F]WC-DZ-F and [^{18}F]Talazoparib

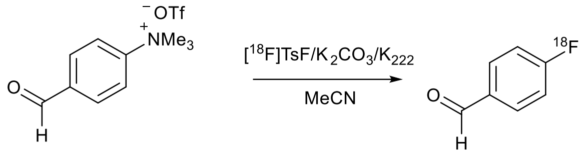

Into a 9 mL Pyrex tube containing K_2CO_3/K_{222} (5.7 mg, 6.4 μmol) and the precursor (4.0 mg, 12.8 μmol) was added [^{18}F]TsF (0.95 GBq) in acetonitrile (0.5 mL). The tube was capped and heated at 102 °C for 8 min. RCC of the reaction is 97% according to radio-TLC analysis of the reaction mixture (Silica gel/ethyl acetate) and 91% according radio-HPLC. 3.3% of total radioactivity was lost to the reaction tube. This reaction was used for the radiosynthesis of [^{18}F]WC-DZ-F.

Note: Too much base (e.g. precursor : K_2CO_3/K_{222} = 2 : 5 w/w) will result in decomposition of the formed [^{18}F]FBAL.

A typical procedure for SPE extraction

The HPLC fraction of a radioactive peak was collected and diluted with water (40 mL) in a 50 mL glass tube. The diluted solution was then passed through an SPE cartridge (Waters C18 light or HLB light) under pressure, applied with a 10 mL syringe, and the cartridge was further rinsed with water (10 mL) and dried with air (10 mL). The radioactivity was then eluted with ethanol in portion (0.1 or 0.2 mL), and the most concentrated portion was diluted with saline to prepare a final dose in 10% ethanol in saline.

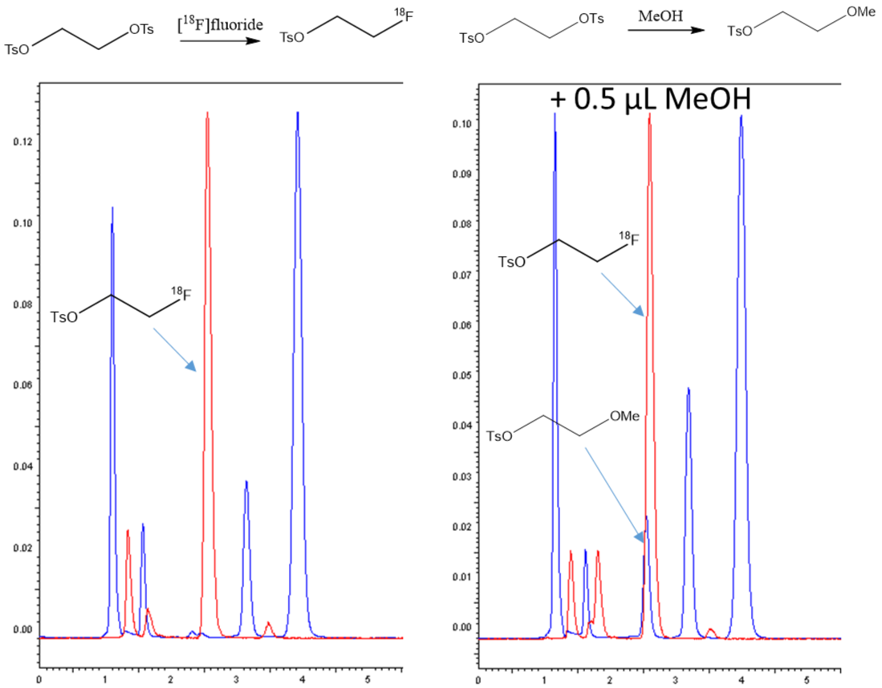

Precursor	2 mg	2 mg
K_2CO_3/K_{222}	3 mg	3 mg
[^{18}F]TsF in MeCN	100 µL	100 µL
MeCN (total volume)	500 µL	500 µL
MeOH	/	0.5 µL
Reaction condition	105 °C/7 min	105 °C/7 min
RCC (radio-TLC)	98%	97%

Figure S34. Effect of Methanol in the conventional nucleophilic radiofluorination. The reaction solution was split and one solution was added with methanol (0.5 µL). After heating 105 °C for 7 min, a peak (the methoxy derivative) was co-eluted with the radioactive peak ([^{18}F]FETs).

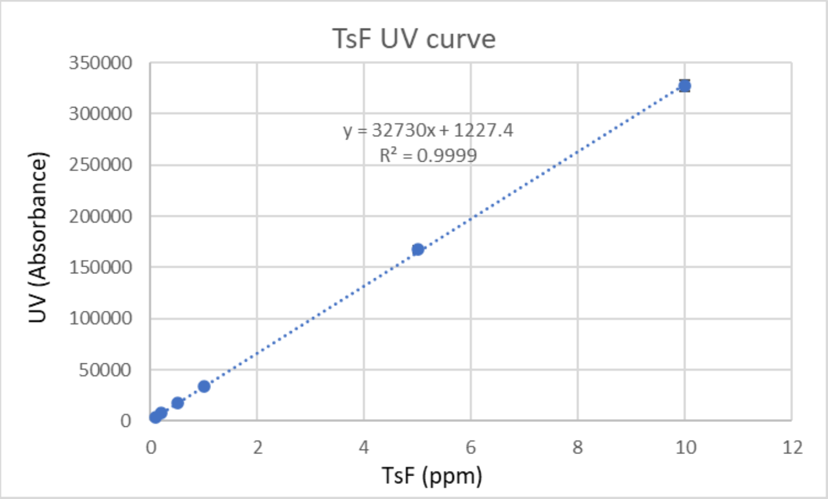

Figure S35. QC curve of TsF (HPLC condition: Altima C18 250 × 4.6 mm, 60% MeCN/40% water/0.1% TFA, 2 mL/min, 228 nm).
